# The Impact of Social Responsibility on the Adoption of Sustainable Development Practices on the risk of stock price collapse

**DOI:** 10.12688/f1000research.188995.1

**Published:** 2026-09-09

**Authors:** Nahla Ibrahim, Abdulaziz Al Naim, Abdulrahman Alomair

**Affiliations:** 1 Accounting Departement, King Faisal University, Al Ahsa, Eastern Province, 31982, Saudi Arabia

**Keywords:** Corporate social responsibility; Sustainable development practices; Stock price crash risk; PLS-SEM; Saudi Arabia; Emerging market.

## Abstract

**Background:**

Corporate social responsibility (CSR) and sustainable development practices have become increasingly important for firms seeking to address stakeholder expectations and improve corporate sustainability. However, their implications for capital-market risk, particularly stock price crash risk, remain unclear in emerging markets. This study examines the relationship between CSR and stock price crash risk and investigates whether the adoption of sustainable development practices mediates this relationship among Saudi-listed firms.

**Methods:**

The study examines 95 non-financial companies listed on the Saudi Exchange in 2023, selected from an initial population of 231 listed companies based on predefined eligibility criteria. CSR and sustainable development practices were measured using multi-item instruments adapted from prior literature. Stock price crash risk was assessed using three commonly employed indicators: CRASHdummy, negative conditional skewness (NCSKEW), and down-to-up volatility (DUVOL). The hypothesized direct and indirect relationships were estimated using variance-based partial least squares structural equation modeling (PLS-SEM) with SmartPLS 4. Measurement-model reliability and validity were assessed before testing the structural paths and mediation effect.

**Results:**

CSR was not significantly associated with stock price crash risk. In contrast, CSR was positively and significantly associated with the adoption of sustainable development practices. Sustainable development practices were also positively associated with stock price crash risk. The indirect relationship between CSR and stock price crash risk through sustainable development practices was statistically significant, supporting the proposed mediating role of sustainable development practices.

**Conclusions:**

The findings indicate that CSR does not have a statistically significant direct relationship with stock price crash risk among Saudi-listed firms. Instead, its association with crash risk operates indirectly through firms’ adoption of sustainable development practices. These results highlight the importance of considering sustainability practices as a potential mechanism linking corporate social responsibility with capital-market outcomes in an emerging-market setting.

## 1. Introduction

With the development of economic thought, the concept of the economic unit as a subsystem within a larger social system that must work to balance with it to ensure survival has evolved, and the goal of maximizing profitability is no longer the primary goal of the economic unit ( Abdelmotaleb, Saha, 2019) Rather, achieving a satisfactory level of profit while meeting a set of social and environmental goals of the economic unit has become the dominant thought of managing investment units and ensuring their survival and continuity in society ( Kim et al., 2012).

The concept of social responsibility is gaining momentum and great interest in all companies as a result of the accumulation of several developments that made this concept go from being considered voluntary and optional practices in dealing with all related parties to compulsory practices that have become a source of achieving excellence, leadership, and increasing competitiveness ( Stephenson, Vracheva, 2016) It supports the survival of companies and ensures their continuity, through the need for the latter to commit and redouble their efforts towards adopting a socially responsible approach ( Zhang et al., 2013) It seeks to build deeper strategic relationships with its stakeholders, so that it can face the challenges it faces today, especially those related to competition, which has introduced social and environmental requirements ( Gololo, 2019).

One of the most widely accepted definitions of social responsibility is that it is a set of context-specific organizational actions and policies that take into account stakeholder expectations and the triple bottom line of economic, social, and environmental performance ( Amidu et al., 2016).

CSR is seen as the way in which companies seek to achieve commercial success in ways that respect ethical values, society, and the natural environment ( Dewi, Dewi, 2017). 

In addition to the previous implications of social responsibility, the company’s commitment to social responsibility improves the company’s reputation ( Park, 2020), The company’s social behavior in a transparent manner can lead the auditor to a good perception of the company’s internal control systems and a good impression of the company’s ethical commitment ( Ni et al., 2019).

Finally, as a result of the disparity in the concept of social responsibility, the effects of social responsibility practices on shareholding companies and society in the contemporary environment ( Manokaran et al., 2018), As a result of ongoing research efforts, it has been widely agreed that practicing social responsibility activities has a number of positive effects ( Boonnual et al., 2017).

These effects include improving the company’s reputation and standing in society, which confirms the society’s recognition of the company and the legitimacy of its existence ( Choi et al., 2018), These effects extend to the stock market in which the company’s shares are traded ( Xu et al., 2014), CSR activities mitigate information asymmetry, increase market liquidity, increase market efficiency, increase the demand for the company’s shares and increase the value of those shares ( Janamrung, Issarawornrawanich, 2015). 

These effects combine and extend to the product market, where the company’s reputation increases, customer confidence increases, and revenue grows, which is reflected in the company’s profitability ( Cherian et al., 2019), In this way, the interests of all stakeholders, including shareholders, are realized, whereas the traditional concept of the company was focused on making profits that maximize value for shareholders only ( Masoud, Halaseh, 2017).

Preserving and protecting the environment has become one of the priorities of modern businesses, with attention to environmental standards being an important point of differentiation between companies ( Ferrero et al., 2016), The application of these standards has become one of the most important requirements for companies to achieve sustainable development, which represents an essential pillar to increase the quality of their information and maximize their profits and the degree of their development and growth ( Oncioiu et al., 2020), It improves environmental performance, improves community relations, reduces costs, and increases profits ( Grimaldi et al., 2020).

In the past few years, companies have increasingly shifted towards sustainable management of their activities in order to achieve a sustainable competitive advantage ( Alsayegh et al., 2020), This trend is characterized by the expansion of corporate goals from primarily economic goals to also include environmental, social, and governance goals ( Agyemang et al., 2021).

Sustainable development is a concept that has received a lot of attention in terms of economic, environmental, and social aspects in order to maximize human well-being in the present without compromising the ability of future generations to meet their needs ( Dogu, Aras, 2019). 

In light of the globalization of financial markets and the technological and information developments witnessed by the world, stakeholders have become more demanding of environmental and social information and communicating financial and non-financial information to stakeholders when formulating corporate strategies and making decisions ( Baah et al., 2021), Sustainability is essential to the long-term success and competitiveness of organizations ( Ching et al., 2017).

Recently, sustainable development standards have become more demanding and organizations and companies are striving to meet stakeholder expectations regarding financial and non-financial metrics ( Aladwan, 2018), The global interest in these standards has created opportunities and challenges, by expanding responsibilities towards governance and sustainable development, as sustainable development is in harmony with corporate theory, which is based on the premise that an economic enterprise is a social organization that has a human role to play and social responsibilities to bear towards all segments of society, including shareholders, workers, creditors, customers, government agencies, trade unions and others, and that decisions made affect all these parties ( Xiaojing et al., 2022).

CRASH Risk is one of the risks facing companies and investors alike, as it affects the future value of the company and thus the wealth of investors ( Asif et al., 2016), Managing the risk of such a collapse is an important consideration for risk management decisions at the corporate level and investment decisions at the investor level ( Noronha et al., 2018), ( Kim et al., 2019) defined CRASH Risk as a phenomenon in which a company’s stock price drops sharply over a short period of time, While ( Yeung and Lento, 2018a) believes that the possibility of a significant decline in the stock price can be observed through the negative skew in the stock’s return distributions over a period of time of trading on it.

On the other hand, there is agreement among some previous studies that CRASH Risk is the possibility of a sharp decline in the market value of a company’s stock ( Harper et al., 2020), CRASH Risk is one of the most important types of risk for investors and companies because it affects the decision-making process ( Fu, Zhifang, 2019), Because at the market level, CRASH Risk explains a large and important part of the change in equity, while at the company level, this risk is a key determinant of the amount of expected returns in the sector in which the company operates ( Al Mamun et al., 2020).

Determinants that increase or reduce the likelihood of a stock price crash include accounting conservatism, earnings management practices, corporate social responsibility, financial reporting quality and comparability ( Chauhan, Kumar, 2019), tax avoidance, quality of accounting accruals, ability to pay short-term liabilities, audit quality, and conformity with financial analysts’ expectations ( Dang et al., 2018).

## 2. Problem of the study

The interest in applying the concept of social responsibility has increased with the increasing role played by private sector organizations as active partners in society, by sponsoring activities related to preserving the environment, improving the quality of life and fighting poverty without affecting investment opportunities and profits. This interest is part of the quest to achieve balance in cooperation between the state, businessmen and society, as it is no longer limited to the private sector and the state is not only concerned with providing public services to the people in the best way, but is obligated to strengthen the bonds of the social contract in several aspects.

This is confirmed by the results of the study ( Olanipekun, et al., 2019), which emphasized that corporate social responsibility is the commitment of companies to use their resources in a way that can be beneficial to society and all stakeholders, and is a reason for creating value in the future and ensuring sustainability and improving corporate performance, and the study ( Manokaran et al., 2018), which recommended that companies should make continuous efforts on a larger scale so that their CSR activities are more in line with the requirements of society.

The novelty of applying the concept in Saudi companies and the lack of a binding mechanism reduces the effectiveness of contributions and makes it difficult to accountability and accountability, as there are still social, environmental, and administrative challenges, and from the perspective of applying the Kingdom’s vision with its development pillars, we believe that strengthening the role of social responsibility in companies is only an application of this vision to achieve sustainable development.

Today, companies face many challenges, the most important of which is the change in the traditional roles of companies, as their role has exceeded from serving customers, providing services, and obtaining profits to playing roles towards improving the livelihoods and well-being of their communities, and although this role usually emerges voluntarily from the company itself, there is an increasing demand by governments for these communities that obliges companies to play their role in the development of the communities through which they make their profits.

The results of several studies ( Angelia, Suryaningsih, 2015) ( Camilleri, 2017) ( Moon, Parc, 2019) have shown that there is widespread adoption of social responsibility and sustainability by many companies globally, and a growing commitment to embedding social responsibility and sustainability in their activities and integrating social responsibility and sustainability into their operational practices.

Although some organizations and companies have a set of systems compatible with the concept of sustainability, the application of sustainability standards is still not mandatory, and so far there is no law that obliges organizations to apply sustainable development standards, and it may be limited to the efforts and initiatives made by organizations and companies in the field of achieving sustainable development and announced on the official websites of organizations and companies or some reports of boards of directors, and in developed countries, interest in applying sustainability standards has increased, but it is still done voluntarily.

The increasing focus on sustainable performance and long-term value creation strategies has led to the need for new reporting and accountability structures that go beyond financial statements into non-financial key performance indicators based on environmental impacts and social responsibility, and the concept of business sustainability has become an important factor in the successful strategic planning of many companies around the world.

This requires the importance of linking the development of society with the development of the private sector, as our society faces many challenges that slow down its development plans, so private sector companies have an opportunity to play the harmonization between the actual development needs and the social responsibility programs in the private sector, and get them out of the umbrella of voluntary efforts to achieve sustainable development and the reflection of this on CRASH Risk of the stock prices of companies listed on the Saudi Stock Exchange through social responsibility programs.

On the other hand, CRASH Risk has become the focus of attention of current and prospective investors, after a large number of corporate scandals that occurred at the turn of the century, as the accounting literature indicated that there is a noticeable increase in studies that deal with aspects related to the risk of future stock price collapse, and one aspect of the increased interest in studies on stock prices is the study of the possibility of a future collapse in the price of these stocks, which has been recognized as CRASH Risk.

Through a review of the annual financial reports of these companies, it was observed that most of them present their financial performance using only economic measures and ignore social, environmental and governance standards, which resulted in a very low level of adoption of sustainable development standards, which may negatively affect CRASH Risk of company stock prices, so it became clear that there is a lack of adoption of sustainable development standards, as they need non-financial information related to environmental, economic, social and governance performance, which is not provided by traditional financial statements in order to make decisions.

Therefore, companies have to have social acceptance through social responsibility because of its positive effects on the adoption of sustainable development practices, which in turn leads to a reduction in CRASH Risk of company stock prices, which is confirmed by the results of the study ( Hunjra et al., 2020), so this study focuses on the following research questions:

- What is the impact of social responsibility on CRASH Risk in listed companies in Saudi Arabia?
- What is the impact of social responsibility on the adoption of sustainable development practices in companies listed on the Saudi Stock Exchange?
- What is the impact of adopting sustainable development practices on CRASH Risk in companies listed on the Saudi Stock Exchange?
- Is there a significant effect of adopting sustainable development practices as a mediating variable on the relationship between social responsibility and CRASH Risk in companies listed on the Saudi Stock Exchange?

## 3. Importance of the study

The study derives its importance by focusing on the following dimensions:

- Investors: Helping investors rationalize their investment decisions by encouraging companies to adhere to their social responsibility and report it transparently, which reflects on their shares in the market and encourages investors to invest in these companies.
- Companies: Encourage companies to increase their market share and increase their legitimacy and compliance with legislation, laws and environmental requirements by obtaining certificates that prove their compliance with the global requirements of environmental management systems and reduce the risks and pressures that they may be exposed to by stakeholders as a result of not fulfilling their social responsibility.
- Regulatory bodies: Contribute to reducing information asymmetry between companies and stakeholders by disclosing all useful and important information to decision makers in accordance with the principles of social responsibility and sustainable development, as social reporting has become one of the most important requirements for listing on the stock exchange.
- Society: Increasing the interest of companies in solving community issues in general as one of the dimensions of contributing to social responsibility and sustainable development, such as solving (unemployment, pollution, transportation, education, health, and others), which requires business organizations to assume their responsibility and play their role efficiently in the development of society **.**

## 4. Literature review

**Regard to social responsibility**, the results of the study ( Dewi, Dewi, 2017), which proved that the application of green banking services strengthens the relationship between corporate social responsibility and corporate continuity, and the study ( Chen et al., 2018), which confirmed that mandatory disclosure of social responsibility changes corporate behavior and generates positive externalities to the detriment of shareholders.

The results of ( Lanis, Richardson, 2012) and ( Zeng, 2016) added that the higher the corporate social responsibility ranking, the lower the involvement of companies in aggressive tax practices, ( Amidu et al., 2016) which indicated that increased CSR activities are associated with increased profit management, and ( Platonova et al., 2018) which indicated that the current CSR activities carried out by Islamic banks in GCC countries can have a long-term impact on their financial performance.

In another context, the results of some previous studies indicated the usefulness and importance of social responsibility practices from the financial point of view of companies, as the results of the study ( Moser, Martin, 2012) found that meeting the requirements of social responsibility reflects positively on the performance and value of companies.

The results of the study ( Manuela and Finn, 2019) confirmed that social responsibility practices have a significant impact on shareholder value in companies represented by stock prices, as well as the results of the study ( Ahmed et al., 2020) indicated that when companies engage in social responsibility activities, this leads to a reduction in CRASH Risk.

The results of the study ( Shuili and Kun, 2021) also proved the important role of the social responsibility report in communicating important information of appropriate value to the market about the social performance of companies in the future.

Also adopted the results of studies ( Suto, Takehara, 2016) ( Xu, et al., 2014) ( Zhi, et al., 2015) in explaining that companies disclose the dimensions of social responsibility works to provide more useful information to stakeholders in rationalizing their decisions, reducing equity risk by reducing the volatility in stock return, which leads to the demand of investors for shares, increasing the liquidity of securities, which is ultimately reflected in maximizing their value and reducing the cost of equity.

**Regard to sustainable development**, the study ( Susanne, 2023), which indicated that the capitalist and allegedly short-sighted focus on maximizing shareholder value may change towards a greater focus on integrating sustainability as an important goal for success in the transition towards sustainable business, and the study ( Alsayegh et al., 2020), whose results indicated that environmental performance and social performance are positively and significantly related to sustainable economic performance, and ( Oncioiu et al., 2020) whose results indicated that corporate social reporting indicators are integrated into financial performance reports with the aim of transforming sustainability into tangible value for all stakeholders, ( Pratiwi et al., 2020) that there is a positive effect between environmental accounting on corporate sustainability.

The study ( Aladwan, 2018) proved that companies are committed to the concept of environmental accounting and its methods in addition to their commitment to social responsibility as part of sustainable development, and the study ( Schreck & Raithel, 2018) which indicated that stakeholders tend to use sustainability information in evaluating corporate performance is a reason for increasing the levels of corporate profitability.

The study ( Schreck & Raithel, 2018), whose results indicated that the tendency of stakeholders to use sustainability information in evaluating the performance of companies is a reason for increasing the levels of profitability of companies, While the study ( Li et al., 2018) emphasized that improving transparency and enhancing stakeholder trust play an important role in improving the value of the company with a positive correlation between the level of disclosure of sustainable development dimensions and the value of the company.

The study ( Noronha et al., 2018), whose results indicated that there is a positive relationship between corporate governance quality and stock market reaction, while ( Tapos, 2017) indicated that there is a correlation between the standard method and quantitative environmental reports to increase documented environmental information.

**Regarding CRASH Risk**, the study ( Butar & Murniati, 2021) emphasized that the adoption of accounting conservatism by companies has a significant impact on reducing CRASH Risk, and the study ( Chae et al., 2020) ( Yin & Tian, 2017), ( Chae et al., 2020) and ( Yin & Tian, 2017), which emphasized that CRASH Risk is the possibility of a sharp decline in the market value of the company’s stock, ( Harmadji et al., 2020), which emphasized that the implementation of sustainability reporting disclosure practices leads to an increase in stock price, and that the likelihood of a future stock price crash risk is influenced by several considerations, including those related to the capital market, While ( He et al., 2019) believes that it is related to the company.

 ( Moradi et al., 2020) indicated that information asymmetry does not affect the relationship between trade credit financing and the risk of stock price collapse, ( Qi & Diao, 2020) indicated that the risk of stock collapse increases significantly when the firm’s operating strategy changes from a specialized operation to a diversified operation.

## 5. Formation of hypotheses

H1: There is a significant effect of social responsibility on CRASH Risk in companies listed on the Saudi Stock Exchange.

H2: There is a significant impact of social responsibility on the adoption of sustainable development practices in companies listed on the Saudi Stock Exchange.

H3: There is a significant impact of adopting sustainable development practices on CRASH Risk in companies listed on the Saudi Stock Exchange.

H4: There is a significant effect of adopting sustainable development practices as a mediating variable on the relationship between social responsibility and CRASH Risk in companies listed on the Saudi Stock Exchange.

## 6. Research model

The research model is based on what was reviewed from previous literature ( Dewi, Dewi, 2017), ( Chen et al., 2018), ( Amidu et al., 2016), ( Platonova et al., 2018), ( Moser, Martin, 2012), ( Ahmed et al., 2020), ( Suto, Takehara, 2016), ( Xu, et al., 2014), ( Zhi, et al., 2015), ( Susanne, 2023), ( Oncioiu et al., 2020), ( Pratiwi et al., 2020), ( Aladwan, 2018), ( Schreck & Raithel, 2018), ( Li et al., 2018), ( Harmadji et al., 2020), and the hypotheses that were formed and formulated, which clarified the direct and indirect effects of the independent variable (social responsibility) (SR) on the dependent variable (CRASH Risk) (RSPC) with the mediating variable (adoption of sustainable development practices) (SDP) as shown in Figure 1.

**Figure 1.**
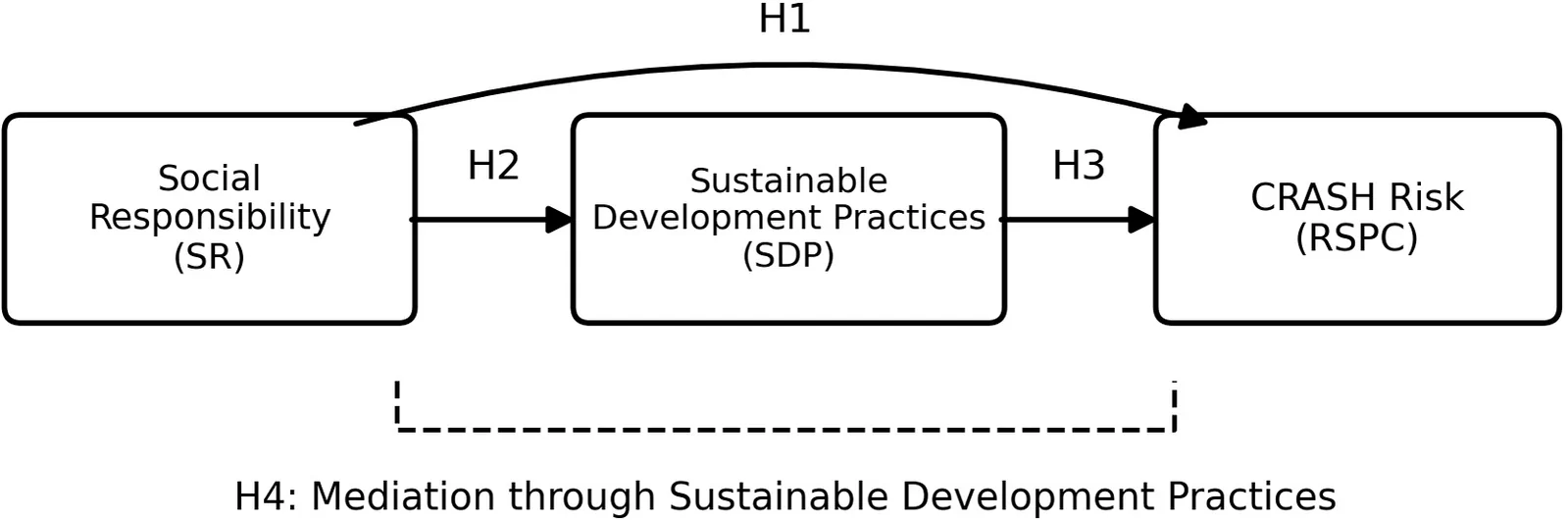
Research model. Source: Authors’ own creation.

## 7. Research methodology

### 7.1 Research community and sample

The study community consists of all Saudi companies registered in the stock market during the period from 2023. The number of these companies by the end of 2023 AD amounted to (231) companies distributed over 9 sectors (petrochemical industries, cement, energy, service facilities, communications, information technology, agriculture, food industries, industrial investment, construction, building materials, real estate development, and multi-investment). The researcher selected the study sample according to the following conditions:

- Excluding the banking sector, the financial services sector, and insurance companies due to their special nature in financial reporting.
- Excluding companies whose financial statements are prepared in a foreign currency so that the financial statements of all sample companies are prepared in the Saudi currency (Saudi riyal).
- Financial reports on the company are available regularly.
- Sufficient data is available to calculate the study variables.
- They have not been subject to delisting, merging, or cessation, meaning that all selected companies are continuing in their activity and have not stopped during the study period.
- The company must have been listed on the Saudi Stock Exchange for more than (10) years, and must not have incurred losses on a regular basis for more than one year.

After applying the previous conditions, the study sample consisted of (95) companies, equivalent to (42.61%) of the total number of joint stock companies registered on the Saudi Stock Exchange ( www.tadawl.com.sa) ( www.mubasher.info) ( www.argaam.com).

### 7.2 Measure variables

7.2.1 Social responsibility questionnaire

Social responsibility (the independent variable) was measured through a questionnaire consisting of (4) axes (prepared by the researchers), where the study ( Amidu et al., 2016) and the study ( Moser, Martin, 2012) were used and reformulated to suit the requirements of the study, by giving (2) two degrees if one of the items is available in the company, and giving (1) one degree if one of the items is available to some extent in the company, and giving (0) degrees if one of the items is not available in the company, then the degrees are collected to represent the degree of social responsibility of the company.

7.2.2 Stock price crash risk

There are many ways to measure the risk of stock price crash, most of which are based on the weekly (daily) returns of the company’s stock, so that each measure reflects company-specific factors rather than a reflection of broad movements in the stock market ( Dang et al., 2018), And the discussion in the present study is based on three ways

- CRASHdummy: The likelihood that the stock’s weekly returns will fall sharply over the next year.

A binary rating scale that takes the value (1) if the stock’s weekly return meets or exceeds the value of 3.09 standard deviations of the stock’s weekly return from its mean value during the fiscal year, with the value of 3. 09 chosen to indicate that there is a 0.1% probability of an abnormal event such as a sharp decline in the stock’s weekly return during the following year according to the normal distribution. The value of 3.09 was chosen to indicate that there is a 0.1% probability of an abnormal event such as a sharp drop in weekly stock returns during the following year according to the normal distribution, indicating a risk of a future collapse of the company’s stock price that takes the value (0) otherwise ( Kim et al., 2016).

- Negative skewness of the stock’s weekly returns (NCSKEW).

It is the most widely used measure in previous studies and depends on the Skewness in the returns on the company’s stock, which represents the asymmetry in the distribution of these returns, and negative values in the skewness indicate that the data is skewed to the left and vice versa, and the value of this measure is calculated by taking the negative amount of the third moment of the daily returns on the stock of the company specified in each period, then converting it to the normal distribution by taking the standard deviation of the daily returns raised to the third power ( Yeung and Lento, 2018b) and this measure takes the following form:

$$\text{NCSKEW}=\frac{N{\left(N-1\right)}^{3/2}\;\Sigma \;D_{j,t}^{3}}{\left(N-1\right)\left(N-2\right){\left(\Sigma \;D_{j,t}^{3}\right)}^{3/2}}$$

Where:

NCSKEW = the negative skewness of the stock’s weekly returns.

D = represents the daily returns on the company’s stock.

n = represents the number of observations during the period.

- Down-Up-to-Volatility (DUVOL)

In this measure, the risk of stock price collapse is estimated through four steps, the first step is to calculate the value of the average weekly market returns calculated Mean, the second step is to determine the returns that are lower than the average calculated in the first step ( Rahman et al., 2019), and the third step is to determine the returns that are higher than the average calculated in the first step ( Rahman et al., 2019), and the standard deviation of these values that are below the average is calculated for each of these weeks, while the third step is to determine the returns that are above the average calculated in the first step, and the standard deviation of these values that are above the average is calculated for each of these weeks, and the fourth step is to calculate the risk of stock price collapse (DUVOL) (Yenug, 2018) as follows:

$$\text{DUVOL}=\mathrm{LN}\;\frac{\left(N_{\text{down}}-1\right)\;\Sigma_{\text{down}}\;R^{2}}{\left(N_{\mathrm{up}}-1\right)\;\Sigma_{\mathrm{up}}\;R^{2}}$$

Where:

N down: is the number of weeks in which the return is less than the average calculated return.

N up: is the number of weeks in which the return is greater than the average calculated return.

7.2.3 Sustainable Development Questionnaire

Sustainable development (the mediating variable) was measured through a questionnaire consisting of (4) axes (prepared by the researchers), where the study ( Alsayegh et al., 2020) was used and reformulated to suit the requirements of the study, by giving (2) two degrees if one of the items is available in the company, and giving (1) one degree if one of the items is available to some extent in the company, and giving (0) degrees if one of the items is not available in the company, then the degrees are added to represent the degree of sustainable development of the company.

### 7.3 Common method variance

Harman’s one-factor test was performed, and its results showed the inadequacy of the one-factor method, as the one-factor matrix explained only 44.070% of the variance, which is less than the threshold value of 50%, so the common method variance did not pose a significant threat to the validity of the measurement ( Podsakoff et al., 2012).

## 8. Data analysis results

Variance-based partial least squares structural equation modeling (PLS-SEM) was adopted through the use of (smartpls4) software, PLS-SEM provides many advantages for researchers, the most important of which are the lack of dependence of the data on the normal distribution, the small sample size, the nature of the latent variables, and the best prediction of research results ( Hair et al., 2014), the model is evaluated in two steps, the first is the evaluation of the measurement model, and the second is the testing of the structural model.

### 8.1 Evaluating the measurement model

It is the first step in variance-based partial least squares structural equation modeling (PLS-SEM), which relies on the use of a structural model with an acceptable level of validity and reliability in preparation for structural path analysis. In this model, (9) items were analyzed within the measurement model, where all items show a strong loading value higher than (0. 70), which is the permissible range ( Hair et al., 2017), so no item was deleted according to the statistical indicators (Cronbach’s alpha, composite reliability, average variance), so no items were deleted from the measurement model according to the statistical indicators (Cronbach’s alpha, composite reliability, average variance) to assess the internal consistency and validity of the scales.

8.1.1 Construction reliability

Table 1 shows the analysis of construct reliability and validity of the measurement model, where Cronbach’s alpha values ranged between (0.793, 0.845), values that exceed the allowed and recommended value (0.70), indicating the internal consistency of the scales, and to emphasize the reliability of the scales, composite reliability was calculated using rho_c, which ranged between (0.863 and 0.907), as it is higher than the recommended value of (0.70) ( Henseler et al., 2016), which confirms the reliability and validity of the scales used in the study, as shown in Table 1 and Figure 2.

**Table 1.** Reliability and validity of scales.

Items	Loadings	Alpha	CR	AVE	VIF
*Social Responsibility*		0.826	0.884	0.655	
SR1	0.837				1.852
SR2	0.811				1.955
SR3	0.767				1.786
SR4	0.821				1.718
*Sustainable Development Practices*		0.793	0.863	0.612	
SD1	0.790				1.365
SD2	0.796				1.913
SD3	0.806				2.046
SD4	0.733				1.504
*CRASH Risk*		0.845	0.907	0.767	
CRASHdummy	0.948				4.231
NCSKEW	0.758				1.548
DUVOL	0.909				3.504

Source: Compiled by the researcher based on the output of smartples 4.

**Figure 2.**
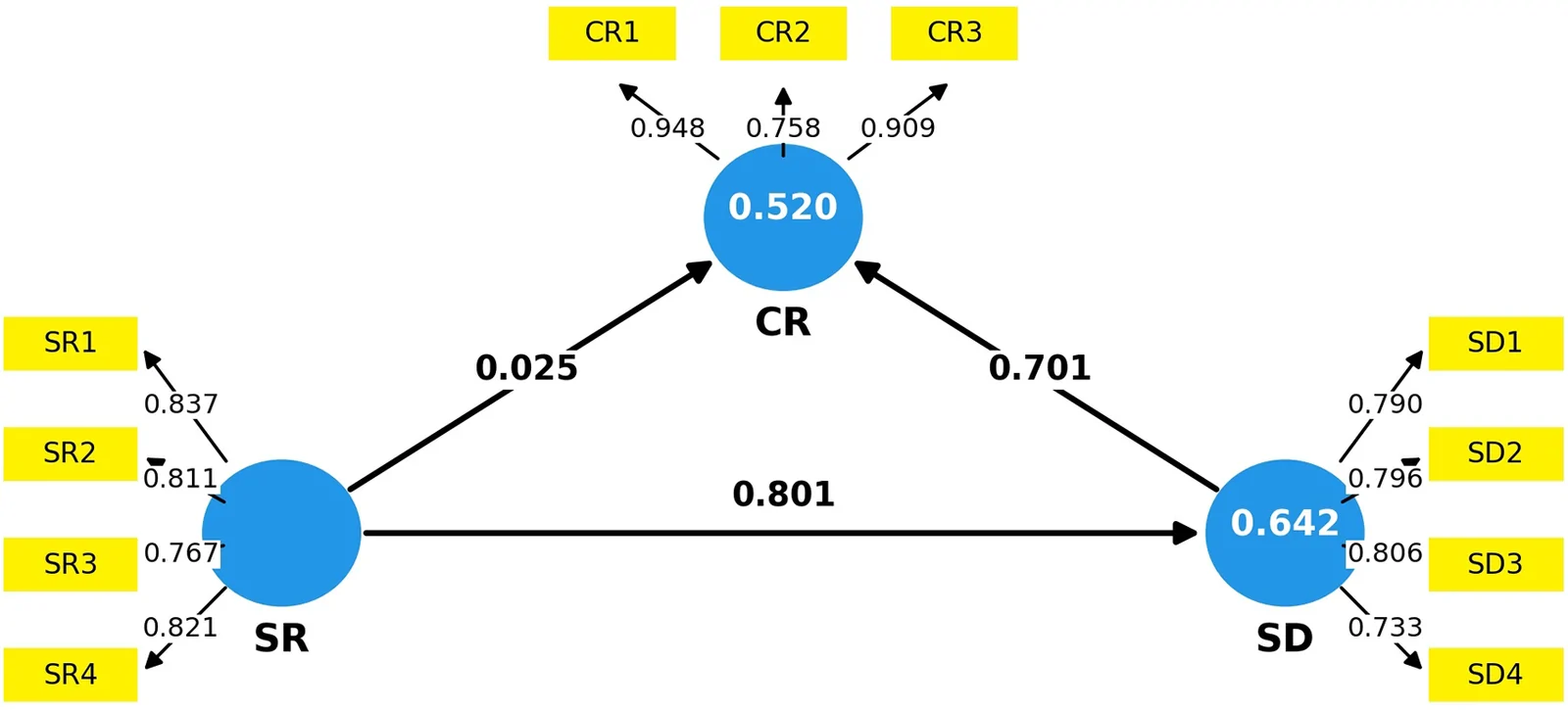
Evaluating the measurement model.

8.1.2 discriminant and convergent validity

To measure the construct validity of the scales, discriminant and convergent validity was used through the average variance extracted index, which ranged between (0.612 and 0.767), which is higher than the acceptable level of (0.50) ( Hair et al., 2017), which confirms the convergent validity of the scales, to evaluate discriminant validity, two indicators were used, the first is the ratio of univariate variance (HTMT) and the second cross-loading (CL) ( Henseler et al., 2016) ( Jha et al., 2023), and it is clear from Table 2 that the HTMT values fall within the permissible range, and from Table 3 the cross-loading values, which indicate the greatest correlation of the phrases with the dimension they form, as it measures the extent to which the phrases diverge from other dimensions ( Hair et al., 2021).

**Table 2.** Univariate Covariance Ratio (HTMT).

Varibles	*SR*	*SD*
*SR*		
*SD*	0.866	
*CR*	*0.680*	0.815

Source: Compiled by the researcher based on the output of smartples 4.

**Table 3.** Cross loading.

Varibles	*SR*	*SD*	*CR*
*Social Responsibility*			
SR1	0.837	0.681	0.525
SR2	0.811	0.580	0.394
SR3	0.797	0.571	0.365
SR4	0.821	0.731	0.573
*Sustainable Development Practices*			
SD1	0.672	0.790	0.869
SD2	0.596	0.796	0.466
SD3	0.626	0.806	0.410
SD4	0.594	0.733	0.361
*CRASH Risk*			
CRASHdummy	0.575	0.692	0.948
NCSKEW	0.407	0.511	0.758
DUVOL	0.543	0.675	0.909

Source: Compiled by the research.

### 8.2 Analyzing the structural model

This step is considered the second step in the evaluation of the measurement model in which the coefficient of determination (R2) and linear interference (VIF) ( Hair et al., 2017), it is clear from Table 1 that the VIF values ranged between (1.365: 4.231), which is less than the allowable value (5) ( Hair et al., 2021). It is also clear from the results of the coefficient of determination (R2) that the amount of variance in the dependent variable (risk of stock price collapse) that can be explained by the independent variable (social responsibility) came (0.520) and the amount of variance in the dependent variable (sustainable development) that can be explained by the independent variable (social responsibility) came (0.642), a value that exceeds (0.50) ( Henseler et al., 2016). That is, social responsibility explains a moderate percentage of the variance in the risk of stock price crash and sustainable development practices.

To test the hypotheses of the study, the researcher performed path analysis of the structural model to assess the statistical significance of the relationships between the study variables, and the significance level (0.05) was determined and Table 4 and Figure 3 show the results of the path analysis.

- The results of the path analysis indicated that social responsibility does not affect the risk of stock price collapse in companies listed on the Saudi Stock Exchange as (B-value = 0.025) (T-value = 0.217) and (P-value > 00.05), so the first hypothesis was rejected.
- The results of the path analysis indicated that social responsibility significantly and positively affects the adoption of sustainable development practices in companies listed on the Saudi Stock Exchange as (B-value = 0.801) (T-value = 20.858) and (P-value < 00.05), so the second hypothesis was accepted.
- The results of the path analysis indicated that the adoption of sustainable development practices significantly and positively affects the risk of stock price collapse in companies listed on the Saudi Stock Exchange as (B-value = 0.701) (T-value = 6.786) and (P-value < 00.05), so the third hypothesis was accepted.

**Table 4.** Path analysis of the study hypotheses.

Hypothesis	Relationship	B-value	SD	t-value	p-value	Decision
H1	SR→CR	0.025	0.116	0.217	0.828	Unsupported
H2	SR→SD	0.801	0.038	20.858	0.000	Supported
H3	SD→CR	0.701	0.103	6.786	0.000	Supported

Source: Compiled by the researcher based on the output of smartples 4.

**Figure 3.**
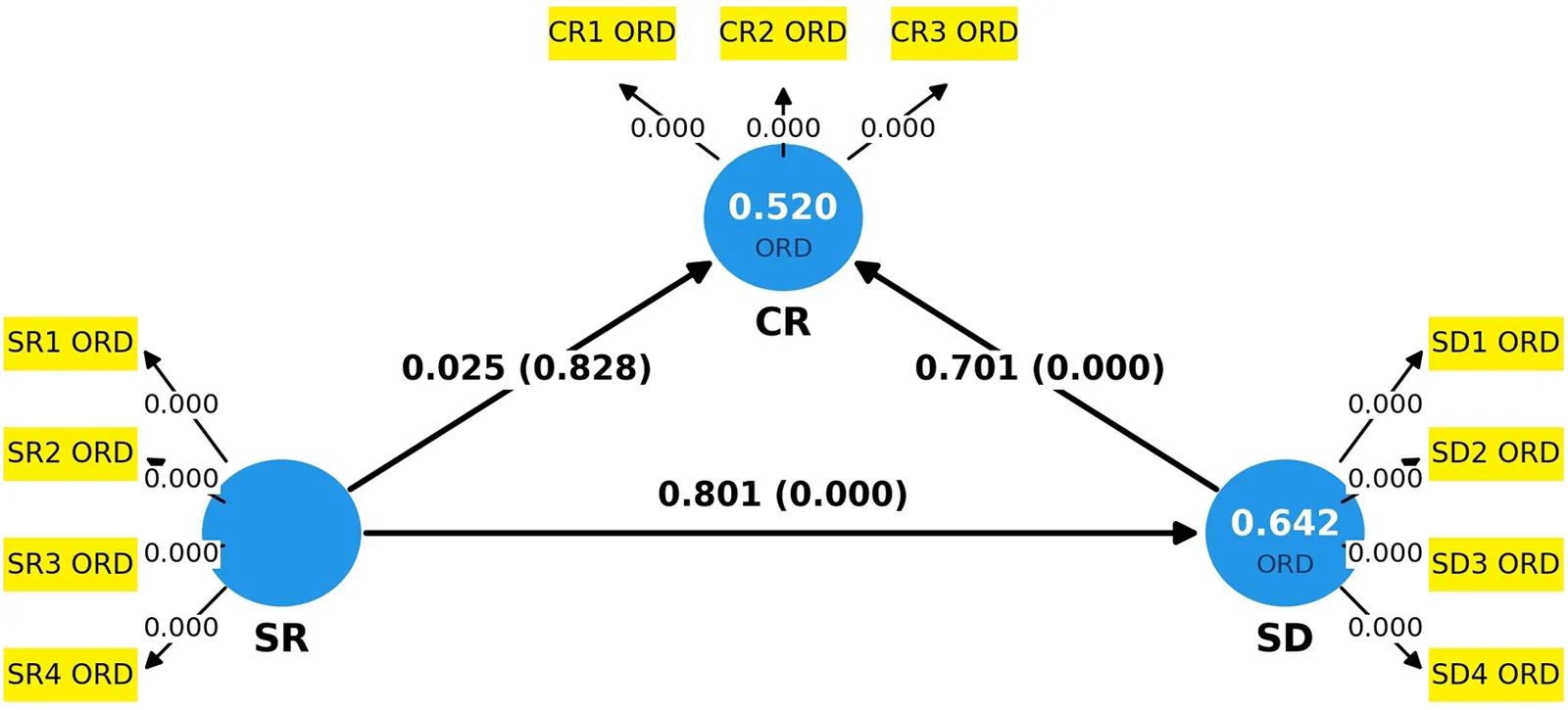
Bootstrapping P-values model.

### 8.3 Analyzing mediation

The fourth hypothesis that sustainable development is a mediating variable in the relationship between social responsibility and the risk of stock price collapse in companies listed on the Saudi Stock Exchange was tested, where the results showed that there is an indirect effect through sustainable development on the relationship between social responsibility and the risk of stock price collapse where (B-value = 0.562) (T-value = 6.249) and (P-value < 00. 05), as well as the total effect of social responsibility on the risk of stock price collapse where (B-value = 0.701) (T-value = 6.786) and (P-value < 00. (B-value = 0.701) (T-value = 6.786) and (P-value < 00.05), but there is no effect of social responsibility on the risk of stock price collapse (B-value = 0.701) (T-value = 6.786) and (P-value < 00.05), but there is no effect of social responsibility on the risk of stock price collapse (B-value = 0. 025) (T-value = 0.217) and (P-value > 00.05) This indicates the effect of the mediator (sustainable development) on the relationship (social responsibility and risk of stock price crash), so the fourth hypothesis was accepted, and this is clear from Table 5.

**Table 5.** Results of the mediation analysis.

Effect	Hypothesis	B-value	SD	t-value	p-value	Decision
Total Effect	SR→CR	0.701	0.103	6.786	0.000	
Direct Effect	SR→CR	0.025	0.116	0.217	0.828	
Indirect effect	SR→SD→CR	0.562	0.090	6.249	0.000	Supported

## 9. Discussion of results

There are many positive effects on companies through the practice of social responsibility, the most important of which is the improvement of the company’s reputation and status in society, and these effects can extend to the stock market in which the company’s shares are traded ( Park, 2020), and preserving and protecting the environment has become one of the priorities of modern companies, which represents a basic pillar to increase the quality of its information and maximize its profits and degree of development ( Boonnual et al., 2017), and managing the risk of stock price collapse is an important consideration for corporate decisions and investment decisions at the investor level ( Noronha et al., 2018). 

Therefore, the current study targeted the extent of the impact of social responsibility on the risk of stock price collapse in companies listed on the Saudi Stock Exchange through the impact of sustainable development based on previous studies that indicated the existence of widespread adoption of social responsibility and sustainability by many companies globally ( Olanipekun, et al., 2019), and the existence of an increasing commitment to include social responsibility and sustainability in their activities and the integration of social responsibility and sustainability in their operational practices ( Manokaran et al., 2018), so companies must have social acceptance through social responsibility because it has positive effects on the adoption of sustainable development practices ( Angelia, Suryaningsih, 2015), which in turn leads to a reduction in the risk of stock price collapse ( Camilleri, 2017).

Based on the theoretical reference and previous studies that refer to the impact of social responsibility and the risk of stock price collapse ( Manuela and Finn, 2019), the first hypothesis was formed, namely that there is a significant effect of social responsibility on the risk of stock price collapse in companies listed on the Saudi Stock Exchange, and the results showed that social responsibility does not affect the risk of stock price collapse in companies listed on the Saudi Stock Exchange and therefore the hypothesis was rejected, and some studies disagree with the result of the hypothesis, including ( Suto, Takehara, 2016), ( Xu, et al., 2014) and ( Zhi, et al., 2015) which indicate that the disclosure of social responsibility dimensions by companies works to provide more useful information to stakeholders in rationalizing their decisions, reducing equity risk by reducing the fluctuation in stock return, which leads to the demand of investors for stocks, and researchers attribute this difference due to the different nature of companies and the environment in which the study was applied.

As for the second hypothesis, which states that there is a significant impact of social responsibility on the adoption of sustainable development practices in companies listed on the Saudi Stock Exchange, the result of the study confirmed the acceptance of the hypothesis, and many studies supported the result of the hypothesis, including ( Alsayegh et al., 2020), whose results indicated that environmental performance and social performance are positively and significantly related to sustainable economic performance, ( Aladwan, 2018) and its results emphasized the commitment of companies to the concept of environmental accounting and its methods in addition to their commitment to social responsibility as part of sustainable development, ( Noronha et al., 2018), whose results indicated a positive relationship between corporate governance quality and the reaction of stock markets.

The third hypothesis emphasized the existence of a significant impact of adopting sustainable development practices on the risk of stock price collapse in companies listed on the Saudi Stock Exchange, and confirmed by previous studies, including ( Alsayegh et al., 2020), whose results indicated that environmental performance and social performance are positively and significantly related to sustainable economic performance, ( Oncioiu et al., 2020) whose results indicated that corporate social reporting indicators are integrated into financial performance reports with the aim of turning sustainability into tangible value for all stakeholders, ( Pratiwi et al., 2020) which indicates that there is a positive impact between environmental accounting on corporate sustainability, ( Schreck & Raithel, 2018) whose results indicate that the tendency of stakeholders to use sustainability information in evaluating corporate performance is a reason for increasing the levels of corporate profitability, ( Harmadji et al., 2020) which emphasized that the application of sustainability reporting disclosure practices leads to an increase in stock price, and that the possibility of the risk of future stock price collapse is influenced by several considerations including those related to the capital market, while ( He et al., 2019) argues that it is related to the company.

Finally, the fourth hypothesis of the study was tested, which states that there is a significant effect of adopting sustainable development practices as a mediating variable on the relationship between social responsibility and the risk of stock price collapse in companies listed on the Saudi Stock Exchange, and the empirical results confirm the existence of a total effect of sustainable development on the relationship between social responsibility and the risk of stock price collapse, this is confirmed by some studies such as ( Alsayegh et al., 2020), whose results indicated that environmental performance and social performance are positively and significantly related to sustainable economic performance, ( Li et al., 2018), whose results indicated that improving transparency and enhancing stakeholder confidence play an important role in improving the value of the company with a positive correlation between the level of disclosure of sustainable development dimensions and the value of the company.

## 10. Conclusions

This study attempted to explore a theory based on the extent of the impact of adopting sustainable development practices on the relationship between social responsibility and the risk of stock price collapse in companies listed on the Saudi Stock Exchange by using the methodology of structural equation modeling of partial least squares based on variance (PLS- SEM), which is considered one of the modern statistical methods to study phenomena and measure the relationships and influences between its variables by building a measurement model that includes a set of indicators indicating it and testing the validity and reliability of this model. SEM is one of the modern statistical methods in studying phenomena and measuring the relationships and influences between their variables by building a measurement model that includes a set of indicators indicating it and testing the validity and reliability of this model, where the process of determining the relationships of influence and impact between the variables that make up the model:- There is no statistical significance of the effect of social responsibility on the risk of stock price collapse in companies listed on the Saudi Stock Exchange at the significance level of 0.05.

- There is a statistical significance of the effect of social responsibility on the adoption of sustainable development practices in companies listed on the Saudi Stock Exchange at the significance level of 0.05.
- There is a statistical significance of the effect of adopting sustainable development practices on the risk of stock price collapse in companies listed on the Saudi Stock Exchange at the significance level of 0.05.
- There is a statistical significance of the effect of adopting sustainable development practices as a mediating variable on the relationship between social responsibility and the risk of stock price collapse in companies listed on the Saudi Stock Exchange at the 0.05 significance level.

## Data Availability

Zenodo: Data supporting the findings of “The Impact of Social Responsibility on the Adoption of Sustainable Development Practices on the Risk of Stock Price Collapse.” https://doi.org/10.5281/zenodo.21917458 ( Ibrahim et al., 2026). The repository contains the underlying dataset used in this study. The dataset comprises 95 non-financial companies listed on the Saudi Exchange in 2023. It includes the measures used to construct corporate social responsibility, sustainable development practices, and stock price crash risk. Corporate social responsibility and sustainable development practices were assessed using multi-item instruments covering their respective dimensions, while stock price crash risk was measured using CRASHdummy, negative conditional skewness (NCSKEW), and down-to-up volatility (DUVOL). The deposited data provide the information necessary to understand the measurement of the study constructs and reproduce the empirical analyses conducted using partial least squares structural equation modeling (PLS-SEM). Extended data are available from the same Zenodo repository: https://doi.org/10.5281/zenodo.21917458 ( Ibrahim et al., 2026). The deposited materials include the underlying dataset and supporting materials relating to the study variables and measurement instruments. The Social Responsibility Questionnaire and Sustainable Development Questionnaire used to construct the study measures are provided as supplementary materials. Any additional supporting documentation, variable descriptions, statistical analysis materials, and replication information are provided in the repository where applicable. Materials created by the authors are available under the licence specified on the Zenodo repository page. Data are available under the terms of the Creative Commons Attribution 4.0 International license (CC-BY 4.0).
