# The Impact of Social Responsibility on the Adoption of Sustainable Development Practices on the Risk of Stock Price Collapse

**DOI:** 10.12688/f1000research.188995.2

**Published:** 2026-11-03

**Authors:** Nahla Ibrahim, Abdulaziz Al Naim, Abdulrahman Alomair

**Affiliations:** 1 Accounting Departement, King Faisal University, Al Ahsa, Eastern Province, 31982, Saudi Arabia

**Keywords:** Corporate social responsibility; Sustainable development practices; Stock price crash risk; PLS-SEM; Saudi Arabia; Emerging market.

## Abstract

**Background:**

Corporate social responsibility (CSR) and sustainable development practices have become increasingly important for firms seeking to address stakeholder expectations and improve corporate sustainability. However, their implications for capital-market risk, particularly stock price crash risk, remain unclear in emerging markets. This study examines the relationship between CSR and stock price crash risk and investigates whether the adoption of sustainable development practices mediates this relationship among Saudi-listed firms.

**Methods:**

The study examines 95 non-financial companies listed on the Saudi Exchange in 2023, selected from an initial population of 231 listed companies based on predefined eligibility criteria. CSR and sustainable development practices were measured using multi-item instruments adapted from prior literature. Stock price crash risk was assessed using three commonly employed indicators: CRASHdummy, negative conditional skewness (NCSKEW), and down-to-up volatility (DUVOL). The hypothesized direct and indirect relationships were estimated using variance-based partial least squares structural equation modeling (PLS-SEM) with SmartPLS 4. Measurement-model reliability and validity were assessed before testing the structural paths and mediation effect.

**Results:**

CSR was not significantly associated with stock price crash risk. In contrast, CSR was positively and significantly associated with the adoption of sustainable development practices. Sustainable development practices were also positively associated with stock price crash risk. The indirect relationship between CSR and stock price crash risk through sustainable development practices was statistically significant, supporting the proposed mediating role of sustainable development practices.

**Conclusions:**

The findings indicate that CSR does not have a statistically significant direct relationship with stock price crash risk among Saudi-listed firms. Instead, its association with crash risk operates indirectly through firms’ adoption of sustainable development practices. These results highlight the importance of considering sustainability practices as a potential mechanism linking corporate social responsibility with capital-market outcomes in an emerging-market setting.

## 1. Introduction

With the development of economic thought, the concept of the economic unit as a subsystem within a larger social system that must work to balance with it to ensure survival has evolved, and the goal of maximizing profitability is no longer the primary goal of the economic unit ( Abdelmotaleb, Saha, 2019) Rather, achieving a satisfactory level of profit while meeting a set of social and environmental goals of the economic unit has become the dominant thought of managing investment units and ensuring their survival and continuity in society ( Kim et al., 2012).

The concept of social responsibility is gaining momentum and great interest in all companies as a result of the accumulation of several developments that made this concept go from being considered voluntary and optional practices in dealing with all related parties to compulsory practices that have become a source of achieving excellence, leadership, and increasing competitiveness ( Stephenson, Vracheva, 2016) It supports the survival of companies and ensures their continuity, through the need for the latter to commit and redouble their efforts towards adopting a socially responsible approach ( Zhang et al., 2013) It seeks to build deeper strategic relationships with its stakeholders, so that it can face the challenges it faces today, especially those related to competition, which has introduced social and environmental requirements ( Gololo, 2019).

One of the most widely accepted definitions of social responsibility is that it is a set of context-specific organizational actions and policies that take into account stakeholder expectations and the triple bottom line of economic, social, and environmental performance ( Amidu et al., 2016).

CSR is seen as the way in which companies seek to achieve commercial success in ways that respect ethical values, society, and the natural environment ( Dewi, Dewi, 2017). 

In addition to the previous implications of social responsibility, the company’s commitment to social responsibility improves the company’s reputation ( Park, 2020), The company’s social behavior in a transparent manner can lead the auditor to a good perception of the company’s internal control systems and a good impression of the company’s ethical commitment ( Ni et al., 2019).

Finally, as a result of the disparity in the concept of social responsibility, the effects of social responsibility practices on shareholding companies and society in the contemporary environment ( Manokaran et al., 2018), As a result of ongoing research efforts, it has been widely agreed that practicing social responsibility activities has a number of positive effects ( Boonnual et al., 2017).

These effects include improving the company’s reputation and standing in society, which confirms the society’s recognition of the company and the legitimacy of its existence ( Choi et al., 2018), These effects extend to the stock market in which the company’s shares are traded ( Xu et al., 2014), CSR activities mitigate information asymmetry, increase market liquidity, increase market efficiency, increase the demand for the company’s shares and increase the value of those shares ( Janamrung, Issarawornrawanich, 2015). 

These effects combine and extend to the product market, where the company’s reputation increases, customer confidence increases, and revenue grows, which is reflected in the company’s profitability ( Cherian et al., 2019), In this way, the interests of all stakeholders, including shareholders, are realized, whereas the traditional concept of the company was focused on making profits that maximize value for shareholders only ( Masoud, Halaseh, 2017).

Preserving and protecting the environment has become one of the priorities of modern businesses, with attention to environmental standards being an important point of differentiation between companies ( Ferrero et al., 2016), The application of these standards has become one of the most important requirements for companies to achieve sustainable development, which represents an essential pillar to increase the quality of their information and maximize their profits and the degree of their development and growth ( Oncioiu et al., 2020), It improves environmental performance, improves community relations, reduces costs, and increases profits ( Grimaldi et al., 2020).

In the past few years, companies have increasingly shifted towards sustainable management of their activities in order to achieve a sustainable competitive advantage ( Alsayegh et al., 2020), This trend is characterized by the expansion of corporate goals from primarily economic goals to also include environmental, social, and governance goals ( Agyemang et al., 2021).

Sustainable development is a concept that has received a lot of attention in terms of economic, environmental, and social aspects in order to maximize human well-being in the present without compromising the ability of future generations to meet their needs ( Dogu, Aras, 2019). 

In light of the globalization of financial markets and the technological and information developments witnessed by the world, stakeholders have become more demanding of environmental and social information and communicating financial and non-financial information to stakeholders when formulating corporate strategies and making decisions ( Baah et al., 2021), Sustainability is essential to the long-term success and competitiveness of organizations ( Ching et al., 2017).

Recently, sustainable development standards have become more demanding and organizations and companies are striving to meet stakeholder expectations regarding financial and non-financial metrics ( Aladwan, 2018), The global interest in these standards has created opportunities and challenges, by expanding responsibilities towards governance and sustainable development, as sustainable development is in harmony with corporate theory, which is based on the premise that an economic enterprise is a social organization that has a human role to play and social responsibilities to bear towards all segments of society, including shareholders, workers, creditors, customers, government agencies, trade unions and others, and that decisions made affect all these parties ( Xiaojing et al., 2022).

A central question is how socially responsible behaviour might be reflected in stock-market risk. When CSR is translated into credible sustainability practices, more transparent reporting may discourage the accumulation of undisclosed adverse information and thereby reduce the likelihood of a sudden price correction. However, implementation costs, symbolic disclosure and heightened investor scrutiny may also be associated with greater observed risk. The direction of the sustainability—crash-risk relationship is therefore not guaranteed by theory.

CRASH Risk is one of the risks facing companies and investors alike, as it affects the future value of the company and thus the wealth of investors ( Asif et al., 2016), Managing the risk of such a collapse is an important consideration for risk management decisions at the corporate level and investment decisions at the investor level ( Noronha et al., 2018), ( Kim et al., 2019) defined CRASH Risk as a phenomenon in which a company’s stock price drops sharply over a short period of time, While ( Yeung and Lento, 2018a) believes that the possibility of a significant decline in the stock price can be observed through the negative skew in the stock’s return distributions over a period of time of trading on it.

On the other hand, there is agreement among some previous studies that CRASH Risk is the possibility of a sharp decline in the market value of a company’s stock ( Harper et al., 2020), CRASH Risk is one of the most important types of risk for investors and companies because it affects the decision-making process ( Fu, Zhifang, 2019), Because at the market level, CRASH Risk explains a large and important part of the change in equity, while at the company level, this risk is a key determinant of the amount of expected returns in the sector in which the company operates ( Al Mamun et al., 2020).

Determinants that increase or reduce the likelihood of a stock price crash include accounting conservatism, earnings management practices, corporate social responsibility, financial reporting quality and comparability ( Chauhan, Kumar, 2019), tax avoidance, quality of accounting accruals, ability to pay short-term liabilities, audit quality, and conformity with financial analysts’ expectations ( Dang et al., 2018).

Saudi Arabia provides a useful setting for examining these issues. Vision 2030, the Capital Market Authority’s corporate-governance reforms and the Saudi Exchange’s ESG Disclosure Guidelines have increased attention to corporate transparency and sustainable business conduct. The 2021 ESG guidance encourages issuers to report material sustainability information; it should be distinguished from binding financial reporting and governance obligations ( Saudi Exchange, 2021; Capital Market Authority, 2023).

## 2. Problem of the study

The interest in applying the concept of social responsibility has increased with the increasing role played by private sector organizations as active partners in society, by sponsoring activities related to preserving the environment, improving the quality of life and fighting poverty without affecting investment opportunities and profits. This interest is part of the quest to achieve balance in cooperation between the state, businessmen and society, as it is no longer limited to the private sector and the state is not only concerned with providing public services to the people in the best way, but is obligated to strengthen the bonds of the social contract in several aspects.

This is confirmed by the results of the study ( Olanipekun, et al., 2019), which emphasized that corporate social responsibility is the commitment of companies to use their resources in a way that can be beneficial to society and all stakeholders, and is a reason for creating value in the future and ensuring sustainability and improving corporate performance, and the study ( Manokaran et al., 2018), which recommended that companies should make continuous efforts on a larger scale so that their CSR activities are more in line with the requirements of society.

The Saudi reporting setting combines binding corporate-governance and periodic disclosure rules with guidance encouraging voluntary ESG reporting. This distinction matters because companies can differ considerably in how they implement and document sustainable development practices, even when subject to similar general listing requirements.

Today, companies face many challenges, the most important of which is the change in the traditional roles of companies, as their role has exceeded from serving customers, providing services, and obtaining profits to playing roles towards improving the livelihoods and well-being of their communities, and although this role usually emerges voluntarily from the company itself, there is an increasing demand by governments for these communities that obliges companies to play their role in the development of the communities through which they make their profits.

The results of several studies ( Angelia, Suryaningsih, 2015) ( Camilleri, 2017) ( Moon, Parc, 2019) have shown that there is widespread adoption of social responsibility and sustainability by many companies globally, and a growing commitment to embedding social responsibility and sustainability in their activities and integrating social responsibility and sustainability into their operational practices.

Although listed firms are subject to financial and corporate-governance disclosure obligations, the Saudi Exchange ESG Disclosure Guidelines are primarily intended to help companies develop sustainability reporting practices. The presence of ESG guidance should not be interpreted as a universal requirement that every issuer publish a standalone sustainability report. This institutional setting provides a reason to investigate variation in corporate practices across firms.

The increasing focus on sustainable performance and long-term value creation strategies has led to the need for new reporting and accountability structures that go beyond financial statements into non-financial key performance indicators based on environmental impacts and social responsibility, and the concept of business sustainability has become an important factor in the successful strategic planning of many companies around the world.

This requires the importance of linking the development of society with the development of the private sector, as our society faces many challenges that slow down its development plans, so private sector companies have an opportunity to play the harmonization between the actual development needs and the social responsibility programs in the private sector, and get them out of the umbrella of voluntary efforts to achieve sustainable development and the reflection of this on CRASH Risk of the stock prices of companies listed on the Saudi Stock Exchange through social responsibility programs.

On the other hand, CRASH Risk has become the focus of attention of current and prospective investors, after a large number of corporate scandals that occurred at the turn of the century, as the accounting literature indicated that there is a noticeable increase in studies that deal with aspects related to the risk of future stock price collapse, and one aspect of the increased interest in studies on stock prices is the study of the possibility of a future collapse in the price of these stocks, which has been recognized as CRASH Risk.

Through a review of the annual financial reports of these companies, it was observed that most of them present their financial performance using only economic measures and ignore social, environmental and governance standards, which resulted in a very low level of adoption of sustainable development standards, which may negatively affect CRASH Risk of company stock prices, so it became clear that there is a lack of adoption of sustainable development standards, as they need non-financial information related to environmental, economic, social and governance performance, which is not provided by traditional financial statements in order to make decisions.

CSR may be associated with crash risk through the sustainability practices adopted by firms. An information-transparency explanation predicts a reduction in crash risk; competing explanations point to implementation costs or more visible adverse information. The research questions below examine these associations without presuming that the proposed intermediate pathway establishes causality.

## 3. Importance of the study

The study derives its importance by focusing on the following dimensions:

- Investors: Helping investors rationalize their investment decisions by encouraging companies to adhere to their social responsibility and report it transparently, which reflects on their shares in the market and encourages investors to invest in these companies.
- Companies: Encourage companies to increase their market share and increase their legitimacy and compliance with legislation, laws and environmental requirements by obtaining certificates that prove their compliance with the global requirements of environmental management systems and reduce the risks and pressures that they may be exposed to by stakeholders as a result of not fulfilling their social responsibility.
- Regulatory bodies: Contribute to reducing information asymmetry between companies and stakeholders by disclosing all useful and important information to decision makers in accordance with the principles of social responsibility and sustainable development, as social reporting has become one of the most important requirements for listing on the stock exchange.
- Society: Increasing the interest of companies in solving community issues in general as one of the dimensions of contributing to social responsibility and sustainable development, such as solving (unemployment, pollution, transportation, education, health, and others), which requires business organizations to assume their responsibility and play their role efficiently in the development of society **.**

For policymakers and market regulators, particularly the Capital Market Authority and Saudi Exchange, the results underline the value of consistent, verifiable and decision-useful sustainability information. Clearer disclosures may help users distinguish substantive business practices from general statements of commitment.

## 4. Literature review

### 4.1 CSR and capital-market outcomes

**Regard to social responsibility**, the results of the study ( Dewi, Dewi, 2017), which proved that the application of green banking services strengthens the relationship between corporate social responsibility and corporate continuity, and the study ( Chen et al., 2018), which confirmed that mandatory disclosure of social responsibility changes corporate behavior and generates positive externalities to the detriment of shareholders.

The results of ( Lanis, Richardson, 2012) and ( Zeng, 2016) added that the higher the corporate social responsibility ranking, the lower the involvement of companies in aggressive tax practices, ( Amidu et al., 2016) which indicated that increased CSR activities are associated with increased profit management, and ( Platonova et al., 2018) which indicated that the current CSR activities carried out by Islamic banks in GCC countries can have a long-term impact on their financial performance.

In another context, the results of some previous studies indicated the usefulness and importance of social responsibility practices from the financial point of view of companies, as the results of the study ( Moser, Martin, 2012) found that meeting the requirements of social responsibility reflects positively on the performance and value of companies.

The results of the study ( Manuela and Finn, 2019) confirmed that social responsibility practices have a significant impact on shareholder value in companies represented by stock prices, as well as the results of the study ( Ahmed et al., 2020) indicated that when companies engage in social responsibility activities, this leads to a reduction in CRASH Risk.

The results of the study ( Shuili and Kun, 2021) also proved the important role of the social responsibility report in communicating important information of appropriate value to the market about the social performance of companies in the future.

Also adopted the results of studies ( Suto, Takehara, 2016) ( Xu, et al., 2014) ( Zhi, et al., 2015) in explaining that companies disclose the dimensions of social responsibility works to provide more useful information to stakeholders in rationalizing their decisions, reducing equity risk by reducing the volatility in stock return, which leads to the demand of investors for shares, increasing the liquidity of securities, which is ultimately reflected in maximizing their value and reducing the cost of equity.

### 4.2 Sustainability reporting and corporate practices

**Regard to sustainable development**, the study ( Susanne, 2023), which indicated that the capitalist and allegedly short-sighted focus on maximizing shareholder value may change towards a greater focus on integrating sustainability as an important goal for success in the transition towards sustainable business, and the study ( Alsayegh et al., 2020), whose results indicated that environmental performance and social performance are positively and significantly related to sustainable economic performance, and ( Oncioiu et al., 2020) whose results indicated that corporate social reporting indicators are integrated into financial performance reports with the aim of transforming sustainability into tangible value for all stakeholders, ( Pratiwi et al., 2020) that there is a positive effect between environmental accounting on corporate sustainability.

The study ( Aladwan, 2018) proved that companies are committed to the concept of environmental accounting and its methods in addition to their commitment to social responsibility as part of sustainable development, and the study ( Schreck & Raithel, 2018) which indicated that stakeholders tend to use sustainability information in evaluating corporate performance is a reason for increasing the levels of corporate profitability.

The study ( Schreck & Raithel, 2018), whose results indicated that the tendency of stakeholders to use sustainability information in evaluating the performance of companies is a reason for increasing the levels of profitability of companies, While the study ( Li et al., 2018) emphasized that improving transparency and enhancing stakeholder trust play an important role in improving the value of the company with a positive correlation between the level of disclosure of sustainable development dimensions and the value of the company.

The study ( Noronha et al., 2018), whose results indicated that there is a positive relationship between corporate governance quality and stock market reaction, while ( Tapos, 2017) indicated that there is a correlation between the standard method and quantitative environmental reports to increase documented environmental information.

### 4.3 Determinants of stock-price crash risk

**Regarding CRASH Risk**, the study ( Butar & Murniati, 2021) emphasized that the adoption of accounting conservatism by companies has a significant impact on reducing CRASH Risk, and the study ( Chae et al., 2020) ( Yin & Tian, 2017), ( Chae et al., 2020) and ( Yin & Tian, 2017), which emphasized that CRASH Risk is the possibility of a sharp decline in the market value of the company’s stock, ( Harmadji et al., 2020), which emphasized that the implementation of sustainability reporting disclosure practices leads to an increase in stock price, and that the likelihood of a future stock price crash risk is influenced by several considerations, including those related to the capital market, While ( He et al., 2019) believes that it is related to the company.

( Moradi et al., 2020) indicated that information asymmetry does not affect the relationship between trade credit financing and the risk of stock price collapse, ( Qi & Diao, 2020) indicated that the risk of stock collapse increases significantly when the firm’s operating strategy changes from a specialized operation to a diversified operation.

Recent studies offer differing conclusions about the relationship between CSR and extreme negative stock returns. Chebbi (2024) examines ESG practices and crash risk in a sustainability and governance setting, while Wu, Zhang and Yang (2024) report a risk-mitigating association for CSR, and Li and Wu (2025) document a positive association in Chinese firms. These mixed findings suggest that reporting quality, institutional context and measurement choices are material to interpretation. The present study adds a Saudi cross-sectional perspective by estimating SDP as an intermediate construct, although it does not identify a time-ordered causal mechanism.

Recent Saudi research further underscores the relevance of governance and sustainable finance. Adam et al. (2026) examine risk-committee arrangements and financial sustainability in Saudi banks, while Al-Matari et al. (2026) explore ESG disclosure in a governance/firm-performance setting. Neither study provides direct evidence for the present crash-risk mediation path, but both illustrate the evolving local research context.

## 5. Formation of hypotheses

H1: Higher CSR is associated with lower stock-price crash risk among Saudi-listed firms.

H2: Higher CSR is associated with greater adoption of sustainable development practices among Saudi-listed firms.

H3: Greater adoption of sustainable development practices is associated with lower stock-price crash risk among Saudi-listed firms.

H4: CSR is associated with lower crash risk through the adoption of sustainable development practices (a negative indirect association).

## 6. Research model

The research model is based on what was reviewed from previous literature ( Dewi, Dewi, 2017), ( Chen et al., 2018), ( Amidu et al., 2016), ( Platonova et al., 2018), ( Moser, Martin, 2012), ( Ahmed et al., 2020), ( Suto, Takehara, 2016), ( Xu, et al., 2014), ( Zhi, et al., 2015), ( Susanne, 2023), ( Oncioiu et al., 2020), ( Pratiwi et al., 2020), ( Aladwan, 2018), ( Schreck & Raithel, 2018), ( Li et al., 2018), ( Harmadji et al., 2020), and the hypotheses that were formed and formulated, which clarified the direct and indirect effects of the independent variable (social responsibility) (SR) on the dependent variable (CRASH Risk) (RSPC) with the mediating variable (adoption of sustainable development practices) (SDP) as shown Figure 1.

**Figure 1.**
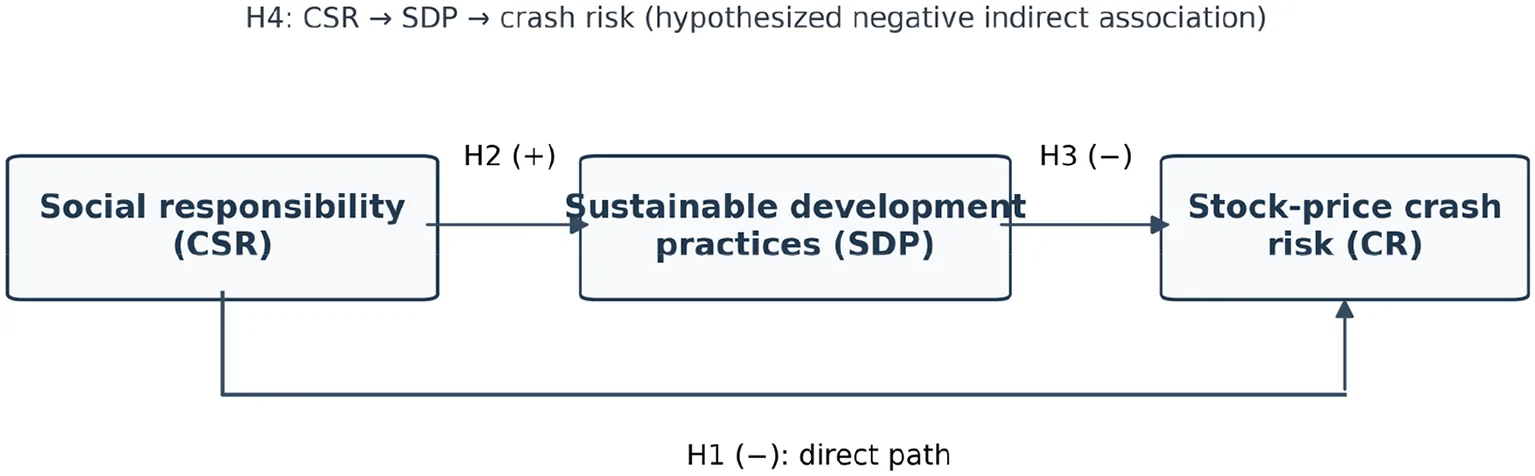
Research model. Source: Authors’ own creation.

Figure 1 illustrates the theoretical direct and indirect paths. The figure is a schematic of the research model.

## 7. Research methodology

### 7.1 Research community and sample

The study examines a cross-section of Saudi-listed firms in 2023. The starting population reported in the journal version was 231 companies, of which 95 non-financial firms met the stated screening criteria. The original description mixes broad sectors with industry subgroups; a verified distribution of the final 95 firms by Saudi Exchange industry is not available in the supporting material. The eligibility rules below were applied to select firms with financial and price information; their restrictive nature may reduce the representativeness of the sample.

- Excluding the banking sector, the financial services sector, and insurance companies due to their special nature in financial reporting.
- Excluding companies whose financial statements are prepared in a foreign currency so that the financial statements of all sample companies are prepared in the Saudi currency (Saudi riyal).
- Financial reports on the company are available regularly.
- Sufficient data is available to calculate the study variables.
- They have not been subject to delisting, merging, or cessation, meaning that all selected companies are continuing in their activity and have not stopped during the study period.
- The company must have been listed on the Saudi Stock Exchange for more than (10) years, and must not have incurred losses on a regular basis for more than one year.

After screening, 95 of 231 companies (41.13%) were retained and 136 were excluded in total. The surviving screening records do not break down the 136 exclusions by criterion. Consequently, the total sample flow is reported below, but a criterion-by-criterion attrition schedule cannot be reproduced from the available records. The restrictions may exclude younger, loss-making or discontinued firms and create selection or survivorship bias.

**Table 1A.** Overall sample flow (2023)

Screening stage	Number of companies
Saudi Exchange companies in stated 2023 population	231
Excluded under the combined eligibility criteria (criteria-specific counts unavailable)	136
Final non-financial company sample	95
Share of initial population retained	41.13%

### 7.2 Measure variables

**7.2.1 Social responsibility questionnaire**

Social responsibility (the independent variable) was measured through a questionnaire consisting of (4) axes (prepared by the researchers), where the study ( Amidu et al., 2016) and the study ( Moser, Martin, 2012) were used and reformulated to suit the requirements of the study, by giving (2) two degrees if one of the items is available in the company, and giving (1) one degree if one of the items is available to some extent in the company, and giving (0) degrees if one of the items is not available in the company, then the degrees are collected to represent the degree of social responsibility of the company.

The CSR instrument reproduced in Appendix A comprises 19 checklist items in four domains: employees (five items), society (five), the environment (five), and customers (four). For each item, the study specifies scores of 2 (available), 1 (available to some extent), or 0 (not available). The four dimensions are represented in the reported measurement model by SR1–SR4. The source materials do not establish whether these forms were completed by company personnel or by researchers examining company documents, and the identity of respondents/coders and independent verification procedures are not documented. Accordingly, we do not attribute the scores to particular executive respondents.

**7.2.2 Stock price crash risk**

The published research model uses CRASHdummy, NCSKEW and DUVOL as indicators of crash risk. These three proxies capture related but different distributional features, with CRASHdummy being binary. The manuscript describes the standard construction from within-year firm-specific weekly returns; the weekly price histories and processing code were not retained in the files available for this revision, so the exact calculations and coding transformations cannot be independently rechecked.

CRASHdummy: An indicator of an unusually large negative firm-specific weekly return within the observed year, rather than a forecast of a crash in a later year.

For an annual firm observation, the conventional CRASHdummy equals 1 if at least one firm-specific weekly return is lower than the firm’s mean weekly return by at least 3.09 within-year standard deviations; otherwise it equals 0. The threshold is a lower-tail (negative), not an upper-tail, event. This definition describes the intended measurement and is not a forecast of a later-year price decline.

- Negative skewness of the stock’s weekly returns (NCSKEW).

NCSKEW quantifies the negative skewness of a firm’s weekly stock-return distribution: larger values represent greater downside asymmetry. With n usable weekly observations and W denoting demeaned firm-specific weekly returns, the conventional measure is:

Where:

NCSKEW=−[n(n−1)^(3/2)×Σ(W3)][(n−1)(n−2)×(Σ(W2))^(3/2)]

NCSKEW = the negative skewness of the stock’s weekly returns.

W denotes demeaned firm-specific weekly returns; n is the number of usable weeks in the annual measurement period.

n = represents the number of observations during the period.

- Down-Up-to-Volatility (DUVOL)

DUVOL compares variation in below-average firm-specific weekly returns with variation in above-average returns. Weeks are split relative to the firm-year average return, squared returns are summed within each subset, and the ratio is adjusted for the number of observations before taking the natural logarithm.

Where:

$$\text{DUVOL}=\ln\left\{\left[\left(n\_\mathrm{up}-1\right)\times \Sigma \_\text{down}\left(W^{2}\right)\right]/\left[\left(n\_\text{down}-1\right)\times \Sigma \_\mathrm{up}\left(W^{2}\right)\right]\right\}$$

n_down and n_up are the numbers of below-average and above-average trading weeks, respectively; W represents demeaned firm-specific weekly returns.

**7.2.3 Sustainable development questionnaire**

Sustainable development (the mediating variable) was measured through a questionnaire consisting of (4) axes (prepared by the researchers), where the study ( Alsayegh et al., 2020) was used and reformulated to suit the requirements of the study, by giving (2) two degrees if one of the items is available in the company, and giving (1) one degree if one of the items is available to some extent in the company, and giving (0) degrees if one of the items is not available in the company, then the degrees are added to represent the degree of sustainable development of the company.

The SDP instrument reproduced in Appendix B has 20 items in four five-item domains: environmental, social, economic and governance practices. Items use the same 0 (not available), 1 (partially available) and 2 (available) coding. The four domains correspond to indicators SD1–SD4 in the published model. The item content is documented, but respondent identity, data-gathering protocol, inter-rater checks and the precise aggregation of raw items into firm-level dimension scores cannot be established from the archived materials.

Both instruments were adapted from prior studies and are included as supplementary materials. The CSR form contains 19 items and the SDP form 20 items, rather than four literal questionnaire questions for each construct. In this article, the four model indicators represent dimensions of these instruments, not necessarily individual raw questions.

### 7.3 Common method variance

Harman’s reported single-factor result of 44.070% is below the commonly cited 50% screening threshold, but this test alone cannot exclude common-method bias. CSR and SDP are based on structured practice-assessment items, whereas the crash-risk metrics are market-return measures; this separation may mitigate a common-source problem for the outcome. No verifiable full-collinearity diagnostic or procedural safeguards were provided in the retained files, and no claim of complete absence of method bias is made.

## 8. Data analysis results

The reported analysis uses SmartPLS 4 to estimate the paths CSR → SDP, CSR → crash risk and SDP → crash risk. The 2023 dataset is cross-sectional at the company level (N = 95); no firm-level financial or governance controls were included in the published structural coefficients. The analyst’s later response notes that 5,000 bootstrap resamples were used, but the saved resampling report was not attached; therefore exact BCa confidence limits and the total-effect inference are not independently verifiable. These paths are interpreted as associations rather than causal effects.

### 8.1 Evaluating the measurement model

The measurement model reports four CSR indicators, four SDP indicators, and three crash-risk proxies (11 indicators in total). The reported loadings exceed 0.70, but loadings and reliability coefficients do not by themselves demonstrate that the three constructs are discriminant, or that a binary crash dummy and two continuous measures are interchangeable reflective indicators.

**8.1.1 Construction reliability**

The reported Cronbach alpha coefficients are 0.826 (CSR), 0.793 (SDP) and 0.845 (crash risk), with composite reliability coefficients of 0.884, 0.863 and 0.907. These values suggest internal consistency in the reported estimation, subject to the measurement-model concerns discussed below.

Figure 2 reproduces the reported loadings and path coefficients schematically; it is not a newly exported SmartPLS output.

**Figure 2.**
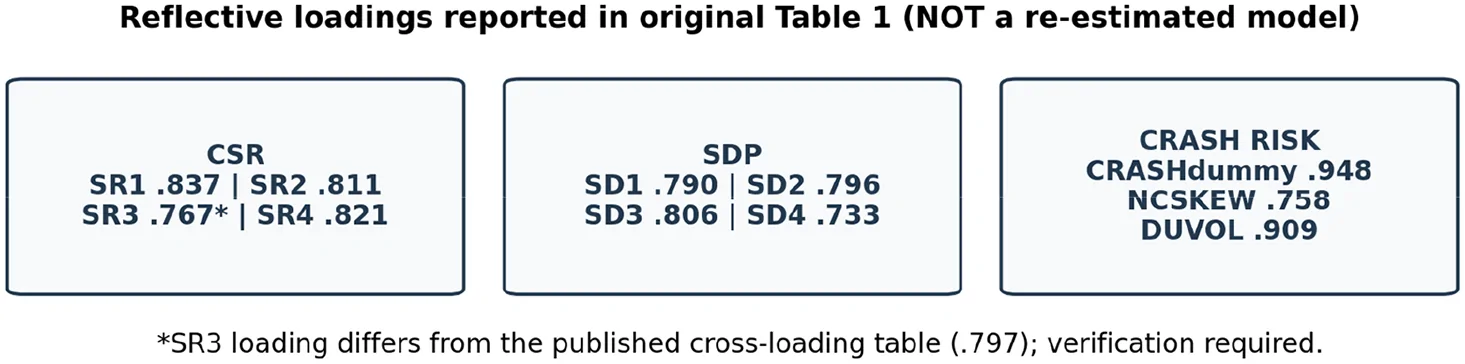
Evaluating the measurement model.

**8.1.2 Discriminant and convergent validity**

The AVE figures range from 0.612 to 0.767, which supports convergent validity under the conventional threshold of 0.50. Discriminant validity is less conclusive. CSR–SDP HTMT is 0.866, falling below 0.90 but exceeding a stricter 0.85 threshold. More importantly, SD1 loads 0.790 on its specified SDP factor but cross-loads 0.869 on crash risk, failing the indicator-level cross-loading criterion. The manuscript therefore does not claim that discriminant validity is fully established. A typographical inconsistency in SR3 was corrected in Table 3 using the value 0.767 reported in Table 1 and the original model diagram; the source SmartPLS file was not supplied for independent verification.

**Table 1.** Reliability and validity of scales.

Items	Loadings	Alpha	CR	AVE	VIF
*Social Responsibility*		0.826	0.884	0.655	
SR1	0.837				1.852
SR2	0.811				1.955
SR3	0.767				1.786
SR4	0.821				1.718
*Sustainable Development Practices*		0.793	0.863	0.612	
SD1	0.790				1.365
SD2	0.796				1.913
SD3	0.806				2.046
SD4	0.733				1.504
*CRASH Risk*		0.845	0.907	0.767	
CRASHdummy	0.948				4.231
NCSKEW	0.758				1.548
DUVOL	0.909				3.504

Source: Compiled by the researcher based on the output of smartples 4.

**Table 2.** Univariate covariance ratio (HTMT).

Varibles	*SR*	*SD*
*SR*		
*SD*	0.866	
*CR*	*0.680*	0.815

Source: Compiled by the researcher based on the output of smartples 4.

**Table 3.** Cross loading.

Varibles	*SR*	*SD*	*CR*
*Social Responsibility*			
SR1	0.837	0.681	0.525
SR2	0.811	0.580	0.394
SR3	0.767	0.571	0.365
SR4	0.821	0.731	0.573
*Sustainable Development Practices*			
SD1	0.672	0.790	0.869
SD2	0.596	0.796	0.466
SD3	0.626	0.806	0.410
SD4	0.594	0.733	0.361
*CRASH Risk*			
CRASHdummy	0.575	0.692	0.948
NCSKEW	0.407	0.511	0.758
DUVOL	0.543	0.675	0.909

Source: Compiled by the research.

### 8.2 Analyzing the structural model

The reported R ^2^ of 0.642 for SDP refers to its association with CSR; the reported R ^2^ of 0.520 for crash risk refers to CSR and SDP together, not CSR alone. Indicator VIF values range from 1.365 to 4.231. The CRASHdummy value of 4.231 is below the stated threshold of 5 but suggests appreciable overlap among crash-risk indicators. The published specification models three heterogeneous crash-risk measures as a reflective latent outcome. As separate-proxy models are not available, their equivalence and sensitivity remain untested limitations of the present analysis.

To test the hypotheses of the study, the researcher performed path analysis of the structural model to assess the statistical significance of the relationships between the study variables, and the significance level (0.05) was determined and Table 4 and Figure 3 show the results of the path analysis.

- The CSR → crash-risk path is small and not statistically significant (β = 0.025, t = 0.217, p = 0.828), so H1 is not supported.
- The CSR → SDP path is positive and statistically significant (β = 0.801, t = 20.858, p < 0.001), consistent with the positive direction proposed in H2.
- The SDP → crash-risk path is positive and statistically significant (β = 0.701, t = 6.786, p < 0.001). It is opposite to the negative direction predicted by H3; therefore H3 is not supported as formulated.

Figure 3 is a schematic redrawing of the reported bootstrap path p-values; it is not a new SmartPLS output.

**Table 4.** Path analysis of the study hypotheses.

Hypothesis	Relationship	B-value	SD	t-value	p-value	Decision
H1	SR→CR	0.025	0.116	0.217	0.828	Unsupported
H2	SR→SD	0.801	0.038	20.858	0.000	Supported
H3	SD→CR	0.701	0.103	6.786	0.000	Significant but opposite to predicted direction

Source: Compiled by the researcher based on the output of smartples 4.

**Figure 3.**
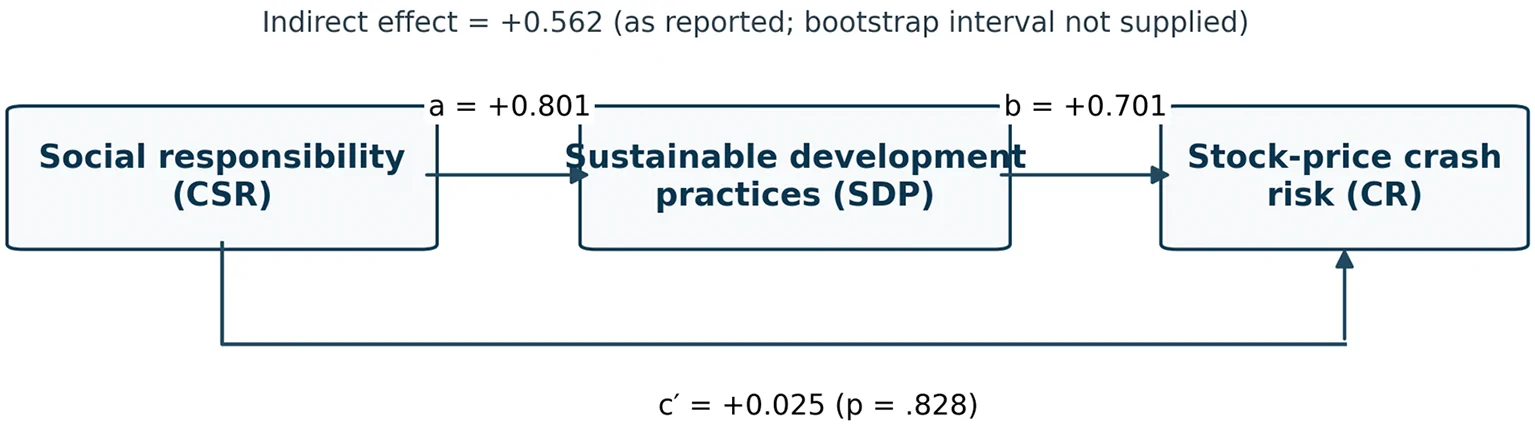
Bootstrapping P-values model.

### 8.3 Analyzing mediation

The reported CSR → SDP path (0.801) and SDP → crash-risk path (0.701) imply an indirect estimate of 0.5615, which rounds to the published 0.562 (t = 6.249, p < 0.001). Adding the reported direct effect of 0.025 (t = 0.217, p = 0.828) produces an arithmetic total of approximately 0.587, rather than the previously reported 0.701, which duplicates the SDP → crash-risk path. The indirect path is positive and significant in the reported analysis, whereas the direct path is not. This is compatible with an indirect-only *statistical* association, but its direction contradicts the pre-specified risk-reduction hypothesis H4. In view of the one-year design and measurement limitations, the result cannot establish temporal or causal mediation.

**Table 5.** Results of the mediation analysis.

Effect	Relationship	Estimate (β)	Reported SE	Reported t	Reported p	Approx. 95% CI†
Total effect	CSR → CR	≈0.587	Not reported	Not reported	Not reported	Not available
Direct effect	CSR → CR	0.025	0.116	0.217	0.828	[−0.202, 0.252]
Indirect effect	CSR → SDP → CR	0.562	0.090	6.249	<0.001	[0.386, 0.738]

*Note*: The total coefficient (0.587) is the rounded sum of the reported direct (0.025) and indirect (0.562) effects; the 0.701 originally displayed as the total was a duplicated path coefficient. Confidence intervals for direct and indirect effects are normal-approximation (estimate ± 1.96 × reported SE), not verified BCa bootstrap intervals. The source materials do not provide a total-effect SE, p-value or bootstrap interval; no values for those fields are inferred.

## 9. Discussion of results

There are many positive effects on companies through the practice of social responsibility, the most important of which is the improvement of the company’s reputation and status in society, and these effects can extend to the stock market in which the company’s shares are traded ( Park, 2020), and preserving and protecting the environment has become one of the priorities of modern companies, which represents a basic pillar to increase the quality of its information and maximize its profits and degree of development ( Boonnual et al., 2017), and managing the risk of stock price collapse is an important consideration for corporate decisions and investment decisions at the investor level ( Noronha et al., 2018). 

Therefore, the current study targeted the extent of the impact of social responsibility on the risk of stock price collapse in companies listed on the Saudi Stock Exchange through the impact of sustainable development based on previous studies that indicated the existence of widespread adoption of social responsibility and sustainability by many companies globally ( Olanipekun, et al., 2019), and the existence of an increasing commitment to include social responsibility and sustainability in their activities and the integration of social responsibility and sustainability in their operational practices ( Manokaran et al., 2018), so companies must have social acceptance through social responsibility because it has positive effects on the adoption of sustainable development practices ( Angelia, Suryaningsih, 2015), which in turn leads to a reduction in the risk of stock price collapse ( Camilleri, 2017).

The insignificant direct association between CSR and crash risk (β = 0.025, p = 0.828) does not mean that CSR has no market implications. It may reflect variation in how substantive CSR commitments translate into practices, incomplete control for firm characteristics, or the restricted sample. With the available data, these are interpretations rather than tested mechanisms.

As for the second hypothesis, which states that there is a significant impact of social responsibility on the adoption of sustainable development practices in companies listed on the Saudi Stock Exchange, the result of the study confirmed the acceptance of the hypothesis, and many studies supported the result of the hypothesis, including ( Alsayegh et al., 2020), whose results indicated that environmental performance and social performance are positively and significantly related to sustainable economic performance, ( Aladwan, 2018) and its results emphasized the commitment of companies to the concept of environmental accounting and its methods in addition to their commitment to social responsibility as part of sustainable development, ( Noronha et al., 2018), whose results indicated a positive relationship between corporate governance quality and the reaction of stock markets.

The positive association between SDP and crash risk (β = 0.701) contrasts with the expected risk-reducing direction and with Chebbi (2024), which reports a negative ESG–crash-risk association among Saudi firms. At the same time, Li and Wu (2025) find a positive CSR–crash-risk relationship in China, suggesting that positive associations are not unprecedented. Differences may arise from disclosure quality, increased investor scrutiny, implementation costs or omitted firm characteristics. None of these pathways is directly identified by our cross-sectional model.

The reported indirect CSR → SDP → crash-risk association is positive (β = 0.562), despite the non-significant direct link. The pattern is consistent with an indirect-only statistical association, rather than evidence that the adoption of sustainability practices reduces crash risk. Since CSR, SDP and the within-year return measures are observed over a common period, neither ordering nor a causal mechanism can be established.

For managers, the findings call for attention to the substance and verifiability of sustainability initiatives rather than an assumption that adopting more initiatives automatically lowers market risk. For investors and the CMA/Saudi Exchange, reporting comparability is important for assessing the information content of CSR and SDP. Further research should use longer panels and evaluate the three crash-risk outcomes separately with relevant firm and market controls.

## 10. Conclusions

This study assesses CSR, sustainability practices and crash-risk proxies for 95 Saudi non-financial listed companies in 2023. CSR has no significant direct association with crash risk (β = 0.025, p = 0.828), while the reported CSR–SDP and SDP–crash-risk paths are positive (β = 0.801 and 0.701). The reported indirect coefficient is also positive (β = 0.562); consequently, the evidence does not support the hypothesised risk-reducing mediation. The total-effect point estimate implied by these coefficients is approximately 0.587, although its inferential statistics are unavailable. The mixed measurement of crash risk, unresolved discriminant-validity issue for SD1, restrictive screening and lack of controls limit the inferences that can be drawn. Future work should employ multiple years, model the three crash-risk proxies separately, and incorporate financial, sector and governance variables.

## Data Availability

Zenodo: Data supporting the findings of “The Impact of Social Responsibility on the Adoption of Sustainable Development Practices on the Risk of Stock Price Collapse.” https://doi.org/10.5281/zenodo.21917458 ( Ibrahim et al., 2026). The repository contains the underlying dataset used in this study. The dataset comprises 95 non-financial companies listed on the Saudi Exchange in 2023. It includes the measures used to construct corporate social responsibility, sustainable development practices, and stock price crash risk. Corporate social responsibility and sustainable development practices were assessed using multi-item instruments covering their respective dimensions, while stock price crash risk was measured using CRASHdummy, negative conditional skewness (NCSKEW), and down-to-up volatility (DUVOL). The deposited data provide the information necessary to understand the measurement of the study constructs and reproduce the empirical analyses conducted using partial least squares structural equation modeling (PLS-SEM). Extended data are available from the same Zenodo repository: https://doi.org/10.5281/zenodo.21917458 ( Ibrahim et al., 2026). The deposited materials include the underlying dataset and supporting materials relating to the study variables and measurement instruments. The Social Responsibility Questionnaire and Sustainable Development Questionnaire used to construct the study measures are provided as supplementary materials. Any additional supporting documentation, variable descriptions, statistical analysis materials, and replication information are provided in the repository where applicable. Materials created by the authors are available under the licence specified on the Zenodo repository page. Data are available under the terms of the Creative Commons Attribution 4.0 International license (CC-BY 4.0).
