# Supplementary material for: The Impact of Social Responsibility on the Adoption of Sustainable Development Practices on the Risk of Stock Price Collapse
Source: F1000Res. 2026 Nov 3;15:1514. Originally published 2026 Sep 9. [Version 2] doi: 10.12688/f1000research.188995.2 (PMC13675628; doi:10.12688/f1000research.188995.2)
Supplement: Peer review history

# Reviewer report: Reviewer response for version 2

**DOI:** 10.5256/f1000research.213463.r538181

**Published:** 2026-11-11

**Authors:** Zainal Abidin

**Affiliations:** 1 Sekolah Tinggi Ilmu Ekonomi Indonesia Jakarta, East Jakarta, Indonesia

This is a relevant and potentially useful study that has improved substantially in response to previous comments. The availability of the underlying data and the authors' efforts to clarify the limitations of the study are particularly welcome.

 Before the manuscript is considered fully robust, I encourage the authors to address the following points:

1. Provide a clearer conceptual distinction between CSR and sustainable development practices.
2. Clarify the construction and measurement procedures for the main study variables.
3. Provide additional justification for combining the three crash-risk proxies into a single construct, and, if possible, report robustness analyses using the proxies separately.
4. Address the remaining discriminant-validity concern associated with SD1.
5. Ensure that the interpretation of the mediation findings reflects the positive direction of the reported indirect association and the non-causal nature of the cross-sectional design.

Addressing these points would further strengthen the methodological transparency and interpretation of this interesting study.

Is the work clearly and accurately presented and does it cite the current literature?

Partly

If applicable, is the statistical analysis and its interpretation appropriate?

Partly

Are all the source data underlying the results available to ensure full reproducibility?

Yes

Is the study design appropriate and is the work technically sound?

Partly

Are the conclusions drawn adequately supported by the results?

Partly

Are sufficient details of methods and analysis provided to allow replication by others?

Partly

Reviewer Expertise:

Business Ethics, CSR and Sustainability

I confirm that I have read this submission and believe that I have an appropriate level of expertise to confirm that it is of an acceptable scientific standard, however I have significant reservations, as outlined above.

# Reviewer report: Reviewer response for version 1

**DOI:** 10.5256/f1000research.208589.r526607

**Published:** 2026-10-16

**Authors:** Musa Ghazwani

**Affiliations:** 1 Jazan University, Jazan, Jazan, Saudi Arabia

The research addresses a highly important topic, and the adopted "Partial Least Squares

 Structural Equation Modeling" (PLS-SEM) methodology is appropriate and sound for testing

 the study's empirical framework. However, there are some minor observations and

 suggestions to help refine and improve the research paper, as follows:

 Introduction& Problem

 1- Briefly frame the background in the context of Saudi Vision 2030 and local corporate

 governance initiatives. Adding a sentence on how Saudi market reforms emphasize

 sustainability will make the topic feel even more grounded in the region.

 2- Enhance transitions between paragraphs to create a smoother narrative flow specifically

 when transitioning from general definitions of Corporate Social Responsibility (CSR) to the

 explanation of stock price crash risk.

 3- Explicitly highlight the theoretical mechanism connecting social responsibility, sustainable

 development practices, and stock price crash risk early in the section. This will help readers

 immediately grasp why sustainable practices act as a mediating pathway.

 4- Provide a brief operational clarification on how mandatory versus voluntary disclosure

 environments in Saudi Arabia impact corporate behaviour, which strongly motivates the

 research questions.

 Importance of the Study

 1- Add a sub-bullet addressing regulatory bodies or policymakers (such as the Saudi

 Capital Market Authority/Tadawul). This will illustrate the practical value of your

 insights for market regulators.

 2- Consider grouping the practical contributions under distinct bold subheadings (e.g.,

 For Investors, For Corporate Management, For Policymakers) for easier reader

 navigation.

 Literature Review

 1- Synthesize the cited literature into thematic sub-headings (e.g., CSR and Financial

 Stability, Sustainability Disclosures, Determinants of Stock Price Crash Risk) to make

 the literature review more structured.

 2- Include a brief concluding summary paragraph at the end of the section that directly

 highlights the specific gap in the Saudi market literature that this study addresses.

 Formation of Hypotheses & Research Model

 1- Ensure the hypothesis statements consistently express the predicted direction of the

 relationships (e.g., whether social responsibility is hypothesized to reduce or increase

 crash risk) to complement the theoretical discussion.

 2- In Figure 1, ensure the layout of arrow connectors cleanly represents the direct and

 indirect mediation paths.

 Research Methodology

 (Note: The variance-based PLS-SEM methodology, sample selection criteria, and variable

 measurement design are appropriate and well-aligned with the research goals.)

 1- Double-check the typographical formatting of mathematical formulas (e.g., NCSKEW

 and DUVOL) to ensure subscripts and fraction bars render cleanly and uniformly

 across all equations.

 2- Briefly describe the demographic/industry profile of the 95 sampled firms in a concise

 summary table or text paragraph to give readers a clearer picture of the dataset.

 Data Analysis Results

 1- Ensure high-resolution figures for the PLS-SEM model outputs (Figures 2 and 3) so

 that loading values and path coefficients are easy to read.

 2- Clean up minor duplications in the textual narrative of Section 8.3 (Mediation

 Analysis) where some output values are repeated, ensuring the statistical values match

 Table 5 cleanly.

 Discussion of Results& Conclusion

 1- Expand slightly on the practical interpretation of the non-significant direct effect (H1)

 vs. the strong mediated pathway (H4). Discussing how sustainable development

 practices bridge this gap adds rich insight.

 2- Contrast your findings with a few recent emerging market studies to demonstrate how

 the Saudi market context compares globally.

 3- Add a short paragraph outlining practical managerial implications and potential

 avenues for future research (e.g., examining longer panel timeframes or specific

 industry sub-sectors).

Is the work clearly and accurately presented and does it cite the current literature?

Yes

If applicable, is the statistical analysis and its interpretation appropriate?

Yes

Are all the source data underlying the results available to ensure full reproducibility?

Yes

Is the study design appropriate and is the work technically sound?

Yes

Are the conclusions drawn adequately supported by the results?

Yes

Are sufficient details of methods and analysis provided to allow replication by others?

Yes

Reviewer Expertise:

ESG and sustainability disclosure

I confirm that I have read this submission and believe that I have an appropriate level of expertise to confirm that it is of an acceptable scientific standard.

# Response

**Published:** 2026-10-23

**Authors:** Abdulaziz Al Naim

**Affiliations:** Accounting, King Faisal University, Al Ahsa, Eastern Province, Saudi Arabia

Comment 1. 

 Response. We inserted a short Saudi institutional-context passage referring to Vision 2030, the CMA’s corporate-governance framework and the Saudi Exchange’s 2021 ESG guidance, taking care not to present guidance-led ESG disclosure as universally mandatory.

 Revision location: Introduction.  Status: Addressed

 Comment 2. 

 Response. We added a linking paragraph explaining why CSR and sustainability implementation might matter for information asymmetry and abrupt price corrections.

 Revision location: Introduction.  

 Comment 3. 

 Response. We now present the transparency-based risk-reduction mechanism alongside the possibility of a positive relationship, distinguishing competing expectations from actual findings.

 Revision location: Introduction; hypotheses.  

 Comment 4. 

 Response. We rewrote the relevant passage to distinguish enforceable financial/governance reporting obligations from Saudi Exchange guidance encouraging sustainability reporting.

 Revision location: Problem statement.  

 Comment 5. 

 Response. The existing regulatory-bodies discussion has been supplemented by a more specific passage on reporting comparability and decision-useful disclosure for the Saudi Exchange and CMA.

 Revision location: Importance; Discussion.  

 Comment 6. 

 Response. The importance section preserves stakeholder categories; in the discussion we now separately articulate managerial and regulatory implications.

 Revision location: Section 3; Section 9.  

 Comment 7. 

 Response. We added a structured introductory bridge and a dedicated “Recent evidence and the research gap” subsection to connect the earlier CSR, sustainability and crash-risk strands. Additional thematic headings can be incorporated without changing the content of the original citations.

 Revision location: Section 4.  Status: 

 Comment 8. 

 Response. We added a final literature-review paragraph distinguishing the study’s intermediate-construct approach from Saudi panel evidence and stating the limits of its cross-sectional contribution.

 Revision location: Section 4.  

 Comment 9. 

 Response. We rewrote H1–H4 in directional form. Crucially, H3 and the expected negative indirect direction are not claimed to be supported merely because the observed coefficients are statistically significant: their signs run counter to the initial prediction.

 Revision location: Section 5; Section 8.2.  

 Comment 10. 

 Response. We supplied a clearer model diagram showing the direct path and the two segments of the indirect path. The conceptual signs are distinguished from reported coefficients.

 Revision location: Figure 1.  

 Comment 11. 

 Response. We replaced ambiguous repeated descriptions with readable mathematical expressions and common definitions. The source calculations need confirmation before representing them as the exact study code.

 Revision location: Section 7.2.2.  

 Comment 12. 

 We reported the documented 95-firm total and described the published eligibility criteria. A valid sector-by-sector frequency table cannot be reconstructed from the available firm roster, so we have not supplied a speculative one.

 Revision location: Section 7.1; Table 1A. 

 Comment 13. 

 The figures have been redrawn in legible resolution from the published coefficients and are explicitly described as schematics of the reported results. We have not described these drawings as original exports from a rerun of SmartPLS.

 Revision location: Figures 2 and 3.  

 Comment 14. 

 The duplicated text was removed. Table 5 now distinguishes direct, indirect and total associations, shows the corrected approximate total (0.587), and labels normal-approximation confidence intervals separately from unverified BCa bootstrap intervals.

 Revision location: Section 8.3; Table 5. 

 Comment 15. 

 The Discussion distinguishes the insignificant direct coefficient from the reported positive indirect pathway. We avoid equating this pattern with a temporally established causal mechanism or with the risk-reduction hypothesis.

 Revision location: Sections 8.3 and 9. 

 Comment 16. 

 Response. We have discussed Saudi evidence from Chebbi (2024), as well as differing international evidence from Wu et al. (2024) and Li and Wu (2025), rather than asserting a uniform ESG effect.

 Revision location: Literature review; discussion.  

 Comment 17. 

 We added implications for managers, investors and regulators and identify multi-year data, proxy-specific models, governance controls and sector heterogeneity as directions for further inquiry. These are future-work recommendations, not analyses falsely claimed to have been carried out.

 Revision location: Sections 9–10. 

# Reviewer report: Reviewer response for version 1

**DOI:** 10.5256/f1000research.208589.r526615

**Published:** 2026-10-15

**Authors:** Nasareldeen Hamed Ahmed Alnor

**Affiliations:** 1 Jouf University, Sakaka, Saudi Arabia

This manuscript examines whether sustainable development practices mediate the relationship between corporate social responsibility (CSR) and stock-price crash risk among Saudi-listed firms. The topic is relevant, and the study addresses an important emerging-market setting. However, substantial clarification and revision are needed before the findings can be evaluated confidently. The main concerns relate to inconsistencies in the reported mediation results, the measurement and validation of the constructs, the short observation period and sample selection, and the interpretation of the positive association between sustainability practices and crash risk. I recommend that the authors address the following points:

 1/ The paths in question are: Corporate Social Responsibility (CSR) → Sustainable Development Practices (SDP) = 0.801, and Sustainable Development Practices (SDP) → Crash Risk = 0.701. Their product is approximately 0.562, which aligns with the reported indirect effect. However, if the direct effect is 0.025, the total effect should be approximately 0.587, rather than the 0.701 reported in Table 5; the reported total effect appears to simply replicate the coefficient value for the path (SDP → Crash Risk). Therefore, the authors should re-verify the SmartPLS output and correct Table 5 and the accompanying text. Furthermore, the direct, indirect, and total effect values ​​derived from the model should be presented—including bootstrap confidence intervals and the number of resamples used—and the type of mediation should be clarified based on the corrected estimates.

 2/ The introduction argues that sustainability practices should reduce crash risk. Yet the reported SDP → crash-risk coefficient is positive and significant (0.701). If higher construct scores consistently represent more sustainability and more crash risk, the result indicates an association with higher, not lower, crash risk. The abstract and conclusion do not adequately address this tension.

 3/ The text alternates between daily and weekly returns, and the CRASHdummy description says returns “meet or exceed” a 3.09-standard-deviation threshold, which does not clearly specify the lower-tail event needed to identify an extreme decline. The NCSKEW and DUVOL formulas are also difficult to interpret in the supplied text, and the calculation steps for DUVOL are repetitive or ambiguous.

 4/ CRASHdummy, NCSKEW, and DUVOL are distinct measures, and one is binary. The paper treats them as indicators of a single latent construct, but it does not explain why this specification is appropriate or whether the indicators are reflective or formative. More seriously, Table 3 reports that SD1 correlates more strongly with crash risk (0.869) than with its intended SDP construct (0.790). That contradicts the text’s claim that items load most strongly on their own constructs.

 5/ Both measures are described as researcher-prepared questionnaires adapted from prior studies, but the paper does not adequately identify the items, dimensions, data sources, respondents, or scoring procedure. It is unclear who completed the questionnaires, how responses were verified, or how the scores were compiled across firms. CSR and sustainability also appear conceptually overlapping: their HTMT value is 0.866, which is below 0.90 but above the more conservative 0.85 threshold. The manuscript also gives different SR3 loadings in Tables 1 and 3 (0.767 versus 0.797).

 6/ The sample covers 95 firms in 2023, but the paper uses causal language and describes SDP as mediating CSR’s effect on crash risk. With a single-year design, it is difficult to establish that CSR precedes SDP and that SDP precedes crash risk. The manuscript also needs to clarify how the annual observation period relates to the return-based measures.

 The authors may also benefit from engaging with broader health-vulnerability literature, including:

 Refer (1)

 Refer (2)

 7/ The structural pathways mentioned appear to overlook firm characteristics and market or governance factors that may be associated with both sustainability practices and stock price crash risk. Without accounting for potential confounding variables, the estimated correlations may not accurately reflect the hypothesized relationships. Therefore, the authors should justify the model's lack of control variables or incorporate appropriate ones where data is available—such as firm size, leverage, profitability, past returns or volatility, industry sector, and governance characteristics. Furthermore, the measurement of these variables should be clarified, and it should be demonstrated whether the main results remain robust and reliable when these variables are included in the model.

 8/ The researchers exclude companies listed on the stock exchange for ten years or less, those incurring recurring losses, and companies that have been delisted, merged, or ceased operations. These restrictions may lead to the exclusion of startups or financially distressed firms—cases that could be relevant when studying crash risk. The paper notes that the 231 companies are distributed across nine sectors but also mentions what appear to be additional sector classifications. Therefore, the authors should explain the rationale for each exclusion, provide a sample flow table detailing the number of companies by criterion and sector, and correct or clarify the sector classifications. They should also discuss potential survivorship and selection biases and, where possible, test whether broader inclusion criteria alter the results.

 9/ The research paper cites the result of Harman’s single-factor test (showing a variance of 44.070%) as evidence that common-method variance does not pose a significant threat. However, this test alone does not rule out common-method bias, particularly when measuring constructs via questionnaires. Consequently, the authors should temper this claim and clarify the procedural steps taken to mitigate common-method bias. It is also advisable—where appropriate—to present results from additional diagnostic tests and to distinguish between variables based on questionnaires and those derived from market data.

 10/ The paper’s explanation of �� ^2^  is unclear: it attributes the variance explained in crash risk to CSR, although the reported model includes SDP as well. The methods section also gives little detail on the model specification and inferential procedure. In addition, the reported VIF for CRASHdummy is 4.231, close to the stated cutoff of 5, but is not discussed.

Is the work clearly and accurately presented and does it cite the current literature?

Yes

If applicable, is the statistical analysis and its interpretation appropriate?

Partly

Are all the source data underlying the results available to ensure full reproducibility?

Yes

Is the study design appropriate and is the work technically sound?

Yes

Are the conclusions drawn adequately supported by the results?

Yes

Are sufficient details of methods and analysis provided to allow replication by others?

Partly

Reviewer Expertise:

Business

I confirm that I have read this submission and believe that I have an appropriate level of expertise to confirm that it is of an acceptable scientific standard, however I have significant reservations, as outlined above.

## References

[1] : Assessing the efficacy of institutional risk management strategies in enhancing financial sustainability: Evidence from Saudi banks. *Corporate Governance and Sustainability Review* .2026;10(4) : 10.22495/cgsrv10i4p4 10.22495/cgsrv10i4p4

[2] : Women Representation in Corporate Leadership and Firm Performance: DoesESG Disclosures Moderate the Relationship?. *Corporate Social Responsibility and Environmental Management* .2026; 10.1002/csr.70988 10.1002/csr.70988

# Response

**Published:** 2026-10-23

**Authors:** Abdulaziz Al Naim

**Affiliations:** Accounting, King Faisal University, Al Ahsa, Eastern Province, Saudi Arabia

Comment 1. 

 We found the arithmetic inconsistency noted by the reviewer. The reported paths (0.801 and 0.701) yield an indirect estimate of approximately 0.562; adding the direct path of 0.025 gives a total of approximately 0.587, not 0.701. Table 5 and Section 8.3 have been corrected. The direct and indirect rows now display approximate 95% intervals calculated from the reported standard errors (estimate ± 1.96 SE) and explicitly identified as such, NOT BCa confidence intervals. The analyst’s reply states that 5,000 bootstrap resamples were used; however, the actual SmartPLS report and total-effect uncertainty statistics were not supplied, so we do not invent a total-effect p-value or state that its BCa interval was verified. A positive significant indirect path with a non-significant direct path is consistent with indirect-only statistical mediation, but not with a causal claim.

 Revision location: Abstract; Section 8.3; Table 5; Conclusion. 

 Comment 2. 

 We agree. The SDP–crash-risk path is positive (β = 0.701), contrary to the hypothesised reduction in risk. We now state its positive direction in the abstract, results, discussion and conclusion, and interpret possible explanations cautiously. H3 and the hypothesised negative indirect sign of H4 are not presented as supported in direction merely because their reported coefficients are statistically significant.

 Revision location: Abstract; Sections 5, 8.2, 9–10; Table 4.  

 Comment 3. 

 We removed the ambiguous forward-looking wording for CRASHdummy and defined the extreme event as a firm-specific return below the annual mean by at least 3.09 within-year standard deviations. The descriptions of NCSKEW and DUVOL use consistent conventional weekly-return terminology, with each symbol defined. The source weekly return histories and preparation code were not supplied; hence the newly clarified conventional formulas are not represented as a separate audit of the original calculations.

 Revision location: Section 7.2.2.  

 Comment 4. 

 We now explicitly state that CRASHdummy, NCSKEW and DUVOL are heterogeneous indicators (one binary, two continuous) and acknowledge that the original reflective specification requires stronger justification. We do not adopt the analyst’s unsupported claim that all three were separately re-estimated, because no such outputs were provided. For discriminant validity, SD1 cross-loads 0.869 on crash risk versus 0.790 on its SDP construct, so the indicator-level criterion is not met. We have withdrawn the blanket statement of adequate discriminant validity and discuss this as a limitation rather than changing the value without the software results.

 Revision location: Sections 8.1, 8.2 and associated table notes

 Comment 5. 

 The two supplementary instruments clarify the previously missing item content: CSR has 19 items across employees, community, environment and customer domains; SDP has 20 items across environmental, social, economic and governance domains. The 0/1/2 rubric is set out in the methodology. We cannot confirm the analyst’s assertion that CFOs and governance/sustainability directors completed the forms, since no respondent roster or survey protocol was supplied; we therefore do not report that as a fact. HTMT = 0.866 is acknowledged as above 0.85 despite being below 0.90. The SR3 value in Table 3 was harmonised to 0.767 based on Table 1 and the reported model graphic; this remains a transcription-level correction rather than independent software validation.

 Revision location: Sections 7.2.1, 7.2.3, 8.1; Table 3; appendices cited. 

 Comment 6. 

 We describe the 2023 observations as cross-sectional firm-level data; annual crash-risk indicators are conventionally derived from returns within a year. The analyst states that each firm-year indicator uses the corresponding 52-week period, but the original return-series files were not attached, and exact trading-week coverage therefore remains unverified. Throughout the manuscript we have avoided assuming that the ordering CSR → SDP → crash risk identifies temporal or causal mediation.

 Revision location: Abstract; Sections 7.1, 7.2.2, 8.3 and 10.  

 Comment 7. 

 We agree that firm characteristics may confound the estimated associations. In the manuscript, we clarify that the published coefficients are from an unadjusted PLS-SEM specification and expressly discuss omitted firm size, leverage, profitability, prior returns, sector and governance measures. The analyst’s written reply asserts that adjusted models preserve significance, but no model coefficients, standard errors or fit information accompany that assertion. For that reason we have not described unverified robustness results as completed, and we present the omission as a limitation.

 Revision location: Section 8 model description; Discussion; Conclusion. 

 Comment 8. 

 We now explain why the original sample excludes financial firms, newer and persistently loss-making companies and firms without continuous financial/price data. We corrected the retained fraction (95/231 = 41.13%) and added the aggregate screening counts. The industry breakdown and numbers excluded at each successive criterion cannot be retrieved from the provided source, and we have not reproduced the analyst’s unsupported claim that Heckman correction or a broadened sample was estimated. Potential selection and survivorship effects are discussed explicitly.

 Revision location: Section 7.1; Table 1A; Discussion; Conclusion. 

 Comment 9. 

 The reported 44.070% one-factor result is described as a limited diagnostic, not proof that common-method bias is absent. We distinguish the structured CSR/SDP instruments from the market-based crash-risk proxies and note what information is missing about procedural precautions. We have not repeated the analyst’s claim that all full-collinearity VIFs are below 3.3 because those outputs were not included, and the published indicator VIF reaches 4.231 (a different quantity that cannot substitute for the unreported full-collinearity diagnostic).

 Revision location: Section 7.3

 Comment 10. 

 We now specify the three PLS-SEM paths, explain R² = 0.642 for SDP and R² = 0.520 for crash risk (the latter jointly explained by CSR and SDP), and discuss the CRASHdummy indicator VIF of 4.231. We distinguish the retained PLS-SEM estimation from the analyst’s unsubstantiated references to panel regression and predictive Q²/f² tests. The later analyst note of 5,000 resamples is identified as reported but not independently verifiable from the saved SmartPLS output.

 Revision location: Sections 8 and 8.2. 

# Reviewer report: Reviewer response for version 1

**DOI:** 10.5256/f1000research.208589.r520521

**Published:** 2026-10-08

**Authors:** Abdalmuttaleb Al-Sartawi

**Affiliations:** 1 Ahlia University, Manama, Bahrain

The paper can be summarized as a study that examines the relationship between social responsibility and stock price crash risk among KSA-listed firms and investigates the mediating role of sustainable development practices in this relationship.

 To the best of my knowledge and judgment, the work is clearly and accurately presented. The citations are fairly current. However, two or three more recent citations could be incorporated to further establish the relevance of the study in light of recent developments in sustainability practices and the regulatory environment in Saudi Arabia.

 The study design and framework are well developed and appropriate given the research objectives.

 Regarding the methodology, further elaboration is needed. The sample selection process requires further clarification to show how the initial population of 231 companies was reduced to the final sample of 95 companies. The number of companies excluded under each criterion should be reported, with justification for key exclusions.

 The conclusions section is adequate. However, the authors are encouraged to discuss some of the possible reasons underlying the findings rather than merely restating the statistical results.

Is the work clearly and accurately presented and does it cite the current literature?

Yes

If applicable, is the statistical analysis and its interpretation appropriate?

Yes

Are all the source data underlying the results available to ensure full reproducibility?

Yes

Is the study design appropriate and is the work technically sound?

Yes

Are the conclusions drawn adequately supported by the results?

Yes

Are sufficient details of methods and analysis provided to allow replication by others?

Partly

Reviewer Expertise:

My research areas include financial accounting & reporting, corporate governance ,sustainable development and sustainable finance.

I confirm that I have read this submission and believe that I have an appropriate level of expertise to confirm that it is of an acceptable scientific standard.

# Response

**Published:** 2026-10-23

**Authors:** Abdulaziz Al Naim

**Affiliations:** Accounting, King Faisal University, Al Ahsa, Eastern Province, Saudi Arabia

Comment 1.

 We expanded the Saudi institutional framing using Saudi Exchange ESG Disclosure Guidelines (2021), the CMA Corporate Governance Regulations (as amended in 2023), and relevant recent studies on governance, sustainability and crash risk. The additional literature is used selectively rather than merely increasing the number of references.

 Revision location: Introduction; literature review; new references.  Status: Addressed in the red-marked draft

 Comment 2.

 The original population was 231 companies and the final 2023 sample was 95 (41.13%). We corrected the percentage and added a brief sample-flow table showing that 136 companies were excluded in aggregate. We cannot provide reliable criterion-specific or sector-specific counts because the corresponding screening register was not included in the archived materials. We have disclosed this limitation rather than creating counts by subtraction or guesswork.

 Revision location: Section 7.1 and Table 1A.  Status: Partial: criterion-specific counts and industry profile pending the screening log

 Comment 3.

 Response. We have rewritten the conclusion and expanded the discussion of possible reasons for the insignificant direct path and positive indirect pathway. We describe symbolic reporting, transition costs, scrutiny and omitted firm characteristics as hypotheses, rather than confirmed explanations. We also clarify the single-year limitation.

 Revision location: Sections 9–10.  Status: Addressed
